# Spatiotemporal quantification of acoustic cell patterning using Voronoï tessellation†

**DOI:** 10.1039/c8lc01108g

**Published:** 2019-02-12

**Authors:** James P. K. Armstrong, Stephanie A. Maynard, Isaac J. Pence, Amanda C. Franklin, Bruce W. Drinkwater, Molly M. Stevens

**Affiliations:** a Department of Materials, Department of Bioengineering, and Institute for Biomedical Engineering, Imperial College London, London, SW7 2AZ, UK; b Department of Mechanical Engineering, University of Bristol, Bristol, BS8 1TR, UK

## Abstract

Acoustic patterning using ultrasound standing waves has recently emerged as a potent biotechnology enabling the remote generation of ordered cell systems. This capability has opened up exciting opportunities, for example, in guiding the development of organoid cultures or the organization of complex tissues. The success of these studies is often contingent on the formation of tightly-packed and uniform cell arrays; however, a number of factors can act to disrupt or prevent acoustic patterning. Yet, to the best of our knowledge, there has been no comprehensive assessment of the quality of acoustically-patterned cell populations. In this report we use a mathematical approach, known as Voronoï tessellation, to generate a series of metrics that can be used to measure the effect of cell concentration, pressure amplitude, ultrasound frequency and biomaterial viscosity upon the quality of acoustically-patterned cell systems. Moreover, we extend this approach towards the characterization of spatiotemporal processes, namely, the acoustic patterning of cell suspensions and the migration of patterned, adherent cell clusters. This strategy is simple, unbiased and highly informative, and we anticipate that the methods described here will provide a systematic framework for all stages of acoustic patterning, including the robust quality control of devices, statistical comparison of patterning conditions, the quantitative exploration of parameter limits and the ability to track patterned tissue formation over time.

## Introduction

Cell organization is a critical component in physiological tissue function, and significant research focus has been invested in engineering *in vitro* systems that can faithfully recreate biological microstructure.^1^ Advances in dielectrophoresis,^2^ three-dimensional (3D) bioprinting,^3^–^5^ and magnetic manipulation^6^–^8^ have provided new opportunities for spatially controlling the development of cell cultures and engineered tissues. Recently, acoustic patterning with ultrasound standing waves has emerged as a potent biotechnology for manipulating single cells and spatially organizing cell populations.^9^,^10^ In this approach, unlabelled cells can be rapidly and dynamically patterned using conventional cell media, culture substrates or biomaterial systems; characteristics that make this technique highly suited to generating complex cell systems. To date, acoustic patterning has been used to generate spheroid cultures,^11^–^14^ engineer complex tissue structures^15^–^18^ and study processes such as vascularization,^19^–^21^ intercellular communication,^22^ tissue development,^23^ cell migration,^24^ natural killer cell activity^25^,^26^ and neurite outgrowth.^27^ This biological versatility arises from the physical mechanisms governing acoustic patterning. In theory, all that is required to achieve patterning is a cell population bearing a difference in density or compressibility with the surrounding medium, and a set of static pressure nodes and antinodes provided by an ultrasound standing wave. Under these conditions, cells acquire a position-dependent potential energy (*U*_rad_) that results in a timeaveraged acoustic radiation force $\left({\vec{F}}_{\text{rad}}\right),$ which moves cells with a density greater than the host medium towards the pressure nodes:^28^

(1) $${\vec{F}}_{\text{rad}}=-\nabla U_{\text{rad}}$$

The magnitude of the acquired energy potential is dependent upon several factors: the mean squared pressure 〈|*p*_0_|^2^〉, the mean squared particle velocity $\langle {\left|{\vec{v}}_{0}\right|}^{2}\rangle ,$ the cell volume (*V*_c_), density (*ρ*_c_) and compressibility (*κ*_c_), and the host medium density (*ρ*_m_) and compressibility (*κ*_m_):^28^

(2) $$U_{\text{rad}}=V_{\text{c}}\left[f_{1}\frac{1}{2}\kappa_{\text{m}}\langle {\left|p_{0}\right|}^{2}\rangle -f_{2}\frac{3}{4}\rho_{\text{m}}\langle {\left|{\vec{v}}_{0}\right|}^{2}\rangle \right]$$

where:

(3) $$f_{1}=1-\frac{\kappa_{\text{c}}}{\kappa_{\text{m}}}$$

(4) $$f_{2}=\frac{2\left(\rho_{\text{c}}/\rho_{\text{m}}-1\right)}{2\rho_{\text{c}}/\rho_{\text{m}}+1}$$

These equations can be used to compute the acoustic radiation force for different acoustic fields. For instance, a single standing wave of the form *p*_0_(*x*) = *P*_0_ cos(*kx*) sin(*ωt*) and unit vector in the *x*-direction $\left({\vec{n}}_{x}\right)$ will produce a one-dimensional field with an acoustic radiation force that is dependent upon the wavelength (*λ*) and speed of sound (*c_m_*) in the host medium:

(5) $${\vec{F}}_{rad}=F_{0}\text{sin}\left(\frac{4\pi x}{\lambda }\right){\vec{n}}_{x}$$

where:

(6) $$F_{0}=\frac{6\pi V_{c}}{\lambda }\left(\frac{f_{1}}{3}+\frac{f_{2}}{2}\right)\frac{P_{0}^{2}}{4\rho_{\text{m}}c_{\text{m}}^{2}}$$

The superposition of a second standing wave in an orthogonal direction $\left({\vec{n}}_{y}\right)$ will create a two-dimensional field, which can be described by an extension of eqn (5) and (6). Here, the acoustic radiation force is proportional to the sum of the squared pressures from each standing wave, where the subscripts *x* and *y* denote the orthogonal directionality for the amplitude and wavelength components of the force distribution:

(7) $${\vec{F}}_{\text{rad}}=F_{\text{o}}^{x}\text{sin}\left(\frac{4\pi x}{\lambda^{x}}\right){\vec{n}}_{x}+F_{\text{o}}^{y}\text{sin}\left(\frac{4\pi y}{\lambda^{y}}\right){\vec{n}}_{y}$$

In practice, however, an acoustic radiation force does not always result in translation of cells to the nodes. The extent of patterning is dependent on the balance between the supplied acoustic radiation force and the forces that act to oppose cell translation. For instance, acoustic patterning can be inhibited by attractive cell-material interactions (*e.g.* with an adherent surface), mechanical agitation, thermal currents or acoustic streaming.^29^ In addition, gravity can oppose the acoustic levitation of cells, while viscous drag becomes an increasingly important consideration as the field moves from solution-based manipulation towards patterning cells in gels.^11^,^16^–^20^,^30^ Overall, the characteristics of the cell, material and field all contribute to the balance of forces experienced by the cell and the final pattern quality. A well-defined patterned array offers: (1) a low proportion of “untrapped” cells, which is essential when attempting to measure bulk functional effects of cell patterning; (2) dense structures with a high degree of cell–cell contact, a critical feature for adhesion and membrane spreading studies; (3) uniformity in structure dimension and geometry, which is vitally important for achieving consistent development and functional properties of cell spheroids and organoid microtissues.^11^–^13^

Given the evident importance of pattern structure and consistency upon the design and outcome of acoustic cell patterning studies, there is a surprising dearth of feature characterization and quality control of the generated cell arrays. The recent, major reports of acoustically patterned cell assemblies have reported only a binary, visual assessment of micrographs to ascertain whether or not a population is patterned.^11^–^14^,^16^–^22^,^24^–^27^,^30^–^34^ Only three of these studies provided characterization beyond basic sizing: Christakou *et al.* indirectly evaluated the cluster “compactness” based on the penetration depth of a fluorescent dye,^26^ Comeau *et al.* used peak-to-peak and peak-to-trough measurements of patterned lines to estimate band spacing and density, respectively,^21^ while Olofsson *et al.* counted both the number of clusters and the number of single cells patterned in each well of a micro-well plate.^13^ In our recent report of acoustically-patterned muscle engineering, we used a Fast Fourier Transform algorithm to identify major frequencies in micrographs of acoustically-patterned myoblasts and define a unidirectional patterning index.^15^ However, such analysis provides a relatively limited assessment of the overall pattern quality, averaged over the entire image, and no quantification of the individual cell structures formed at the acoustic pressure nodes. In principle, it is actually relatively straightforward to characterize well patterned arrays, as these typically have tightly packed structures that can be identified using pixel intensity thresholding. However, this direct approach cannot be readily applied to the analysis of loosely packed structures that do not exhibit a clearly identifiable perimeter. Thus, a more sophisticated form of image analysis is required to make fair comparisons across different patterning conditions.

Here, we demonstrate the quantification of acoustically-patterned cell systems using Voronoï tessellation, an image analysis tool that has previously been used to compute atomic packing,^35^ simulate the cosmic evolution of galaxies^36^ and analyze the clustering of cell membrane proteins.^37^ This algorithm uses localized points in space known as “seeds” to generate a set of tessellated polygons, which each contain a single seed and encompass the points in space closer to its own seed than any other seed.^37^ In this case, the seeds corresponded to high intensity pixels arising from the fluorescence of cells that had been acoustically patterned into clusters under different experimental conditions (pressure amplitude, ultrasound frequency, cell concentration and biomaterial viscosity). We used Voronoï tessellation maps to evaluate patterning using a series of metrics (*e.g.* cluster density, ratio, area, number, barycenter), which allowed us to compare pattern quality and consistency in an impartial, quantitative and statistical manner. Moreover, we extended this algorithm to the analysis of spatiotemporal processes: the translation of cells exposed to an ultrasound field as well as the migration and proliferation of cells acoustically patterned on an adherent surface. The studies detailed in this report highlight some of the most relevant and practical applications of Voronoï tessellation for acoustic cell patterning. However, the versatile nature of this approach should enable application across different patterning systems and experimental conditions, from quantitative characterization and tracking of cell arrays to monitoring device performance or predicting the characteristics of untested cell systems.

## Experimental

### Acoustic patterning devices

All reagents were acquired from Thermo Fisher Scientific, unless otherwise stated. Two patterning devices were constructed with designs similar to those used in previous studies.^15^,^38^,^39^ A 12 mm thick acrylic base plate was laser cut with an inner chamber of either 38.5 × 38.5 mm (device 1) or 10.5 × 10.5 mm (device 2) flanked by four outer chambers containing affixed piezotransducers with wrap-around electrodes (NCE51 12 × 4 × 1 mm, Noliac) (Fig. S1†). Electrical wires were soldered to the piezotransducer electrodes and an acetate sheet was glued to the acrylic base to allow all the chambers to be filled with filter-sterilized dH_2_O. Opposing piezotransducers were wired as a pair, with each pair connected to a TG120 20 MHz Function Generator (Aim TTi). Continuous sine waves were used to create cell assemblies: linear arrays were produced using a single piezotransducer pair driven at 2.1 MHz, while clusters were formed by driving both transducer pairs independently using two unsynchronized function generators set at the same frequency (2.1 × 2.1 MHz or 6.7 × 6.7 MHz). Note that in each case, the measured frequencies showed a small fluctuation (approximately ± 0.05 MHz) hence the signals generated were uncorrelated. The load voltages reported herein refer to the peak-to-peak voltage (V_pp_) measured by an IDS6052-U Digital Storage Oscilloscope (Iso-Tech) during operation of the device (75% of the voltage input by the function generator). The pressure field was measured for acoustic patterning device 1 using a calibrated fibre-optic hydrophone (Precision Acoustics Ltd) mounted on a motorized *X*–*Y* stage. A raster scan was performed across a 6 × 6 mm scan area with a 50 μm step size. The output voltage was sampled at 50 MHz and converted to the frequency domain *via* a fast Fourier transform. Two peaks were identified in the frequency domain, corresponding to the two orthogonal standing waves. Following eqn (7), the pressure amplitudes were extracted at these frequencies, squared then summed to calculate the mean squared pressure. The impedance of acoustic patterning device 1 was characterized between 1–4 MHz using a CypherGraph C60 (Cypher Instruments).

### Patterning in solution

C2C12 myoblasts (ATCC) were cultured in expansion medium comprising high glucose Dulbecco's Modified Eagle's Medium (HG-DMEM) with 1% (v/v) penicillin/streptomycin and 20% (v/v) fetal bovine serum. The myoblasts were harvested, fixed in 4% (w/v) formaldehyde and then membrane stained using wheat germ agglutinin conjugated to Alexa Fluor 633 (WGA-633). 2 mL of stained myoblasts were transferred to a 35 mm tissue culture dish pre-coated with 2 mL autoclaved 2% (w/v) UltraPure Agarose 1000 and patterned into lines or clusters using device 1. 4 × 4 arrays of cell clusters were imaged after 5 min of patterning using a 5× objective lens on an SP5 inverted confocal fluorescence microscope (Leica). Alternatively, time-lapse images were captured every 1 s, with the ultrasound field applied after 5 s of imaging. Larger arrays of cell clusters were generated by stitching a 7 × 7 grid of images captured using a 5× objective lens on an Observer 2.1 wide field microscope.

### Migration of patterned cells

A 10 × 10 mm glass coverslip (Agar Scientific) was coated in 100 μg mL^−1^ poly-d-lysine (Sigma) and then 10 μg mL^−1^ laminin (from Engelbreth-Holm-Swarm murine sarcoma basement membrane, Sigma) to provide an adherent substrate for cell migration. A 2 mL solution of 2.5 × 10^5^ C2C12 myoblasts that had been transfected with lentiviral particles (LVP340, Amsbio) to express GFP were harvested and patterned on the coated coverslip in a 35 mm agarose-coated tissue culture dish using device 1 (45 min, 2.1 MHz, 15 V_pp_). The glass slide was washed once in culture medium to remove non-adherent cells and then placed in a 4-well chamber slide (LabTek). At this stage, 300 μL of freshly-thawed 50% (v/v) Matrigel Matrix (Corning) was added to one of the samples and gelled for 15 min at 37 °C. The patterned cells were cultured in 400 μL of culture medium supplemented with 20 mM HEPES. A 3 × 3 array of clusters was imaged every 5 min using a 10× objective lens on an Observer 2.1 wide field microscope (Zeiss).

### Patterning in hydrogels

The hydrogel synthesis and gelation protocols have been previously published.^40^ Briefly, 20 kDa 8-arm poly(ethylene)glycol (PEG) amine (JenKem) was functionalized with activated 5-norbornene-2-carboxylic acid (Alfa Aesar) to produce 8-arm PEG norbornene. Myoblasts were fixed and membranestained with WGA-633, as before, and then patterned in 5% (w/v) PEG norbornene, 0.05% (w/v) Irgacure, 0–3% (w/v) 200 kDa PEG dopant (Sigma Aldrich) and PEG dithiol crosslinker (Sigma Aldrich), all diluted in phosphate buffered saline (PBS) with a thiol : norbornene ratio of 0.8 : 1. Viscosity creep tests were performed at room temperature using an AR 2000ex rheometer (TA Instruments) using 300 μL of precursor solution, a 25 mm plate, a 500 μm gap and 1 Pa stress. Patterning was performed in the central cavity of device 2 (2.1 × 2.1 MHz, 15 V_pp_, 7 min) with the solution crosslinked for the final 2 min using ultraviolet light (365 nm, 6 mW cm^−2^). The crosslinked hydrogels were removed from the device and maintained in PBS overnight. This procedure generated hydrogels containing a large array of patterned clusters with pattern quality only reduced at the very edges of the material. We used a 5× objective lens and confocal fluorescence microscopy to capture 3 × 3 arrays at the center of the hydrogel, with the smaller number of imaged clusters due to the increased node separation caused by the material swelling. We used the same protocol, without any dopant, to produce patterned PEG hydrogels for imaging using wide field microscopy with either bright field, differential interference contrast (DIC) or phase contrast filters.

### Voronoï tessellation analysis

The following protocol was used for all images, unless stated otherwise. For a full process chart and software links, refer to Fig. S2.† Fluorescence micrographs were processed using FIJI software (open source) with the format converted to 8-bit and the minimum brightness raised to remove any background noise. Seeds were identified from high intensity pixels using ThunderSTORM (open source FIJI plugin) with input pixel size and non-maximum suppression: note that large cells could contain more than one seed. Voronoï tessellation diagrams were generated from the pixel map using SR-Tessler software (open source).^37^ The corr2 function MATLAB R2015a (The MathWorks, Inc.) was used to compute 2D correlation coefficients between patterned cell array images and clusters generated by varying the seed density and cut distance. All solution and hydrogel arrays were analyzed using a seed density factor of 2, a cut distance of 1 × 10^5^ nm and no restrictions placed on the minimum size or number of seeds. This analysis produced the number, area, barycenter and density of the clusters, while the proportion of clustered seeds was calculated by taking into account the total number of seeds (using a seed density factor of 0). Time-lapse videos were cropped to 1400 × 1400 μm (live patterning) and 1170 × 1170 μm^2^ (live migration), with the latter analyzed using a seed density factor of 1.5. Note that Voronoï tessellation analysis of bright field and phase contrast images required an image intensity inversion prior to seed identification.

## Results

**Fig. 1:**
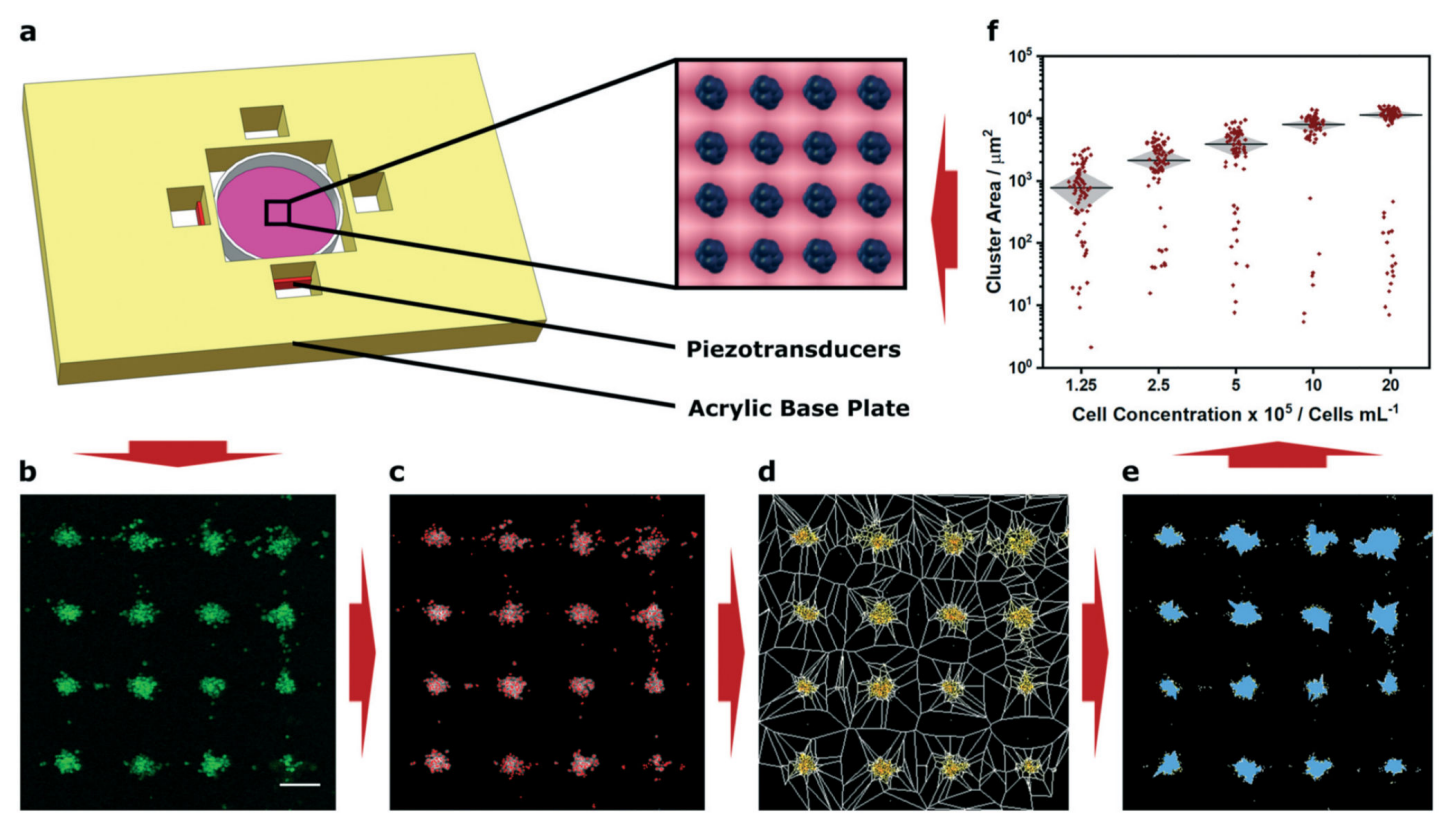
Demonstration of acoustic cell patterning and Voronoï tessellation analysis. (a) A custom-built acoustic patterning device was assembled with four orthogonal transducers surrounding a central cavity enclosing a 35 mm tissue culture dish holding a cell suspension. The piezotransducers were driven at resonance frequency to generate ultrasound standing waves capable of producing clustered arrays of cells (blue, inset). (b) Confocal fluorescence microscopy was used to capture micrographs of clusters of cells (green) after 5 min of acoustic patterning. Scale bar = 200 μm. (c) High intensity pixels were used to determine seeds (red markers), (d) which were used to construct a Voronoï tessellation map. (e) A threshold of the seed density was then used to identify cell clusters (blue). (f) This approach enabled quantitative assessment under different experimental conditions, for example, the measurement of cluster area at different cell concentrations. This information can then be used, if necessary, to inform the parameters of any future acoustic patterning. The data shown was collected from four separate images per group and plotted as median ± interquartile range. For a full analysis of the effect of cell concentration, refer to Fig. S5.†

We fabricated an acoustic patterning device with four 12 mm piezotransducers arranged as orthogonal pairs around a central cavity. This cavity was used to house a 35 mm tissue culture dish containing a suspension of murine myoblasts (C2C12 line) that had been membrane stained with a fluorescent dye (Fig. 1a). By driving the piezotransducers at a frequency close to their primary resonance (2.1 MHz) we were able to translate the cells towards the acoustic pressure nodes and form clustered cell populations with defined periodicity (Fig. 1b). The region of clustered cells was located at the center of the petri dish, the intersection of the two ultrasound standing waves, and typically contained around 300 uniform clusters (Fig. S3†). We imaged these patterned arrays using confocal fluorescence microscopy, and then applied Voronoï tessellation protocols adapted from super-resolution microscopy.^37^ First, we defined a coordinate map of “seeds”, corresponding to the high intensity pixels arising from the cell fluorescence (Fig. 1c). We used this map to segment the image into multiple tessellating polygons, bearing edges equidistant from the nearest two seeds (Fig. 1d). These Voronoï diagrams exhibited distinct and predictable geometry, with distinct regions of densely-packed seeds encased in small polygons surrounded by sparsely-packed seeds in large polygons. From these tessellations, we were able to define discrete cell clusters by applying a threshold on the first-rank seed density factor and cut distance (Fig. 1e). We show that a two-dimensional cross correlation coefficient can be used as an unbiased metric to determine optimal threshold parameters for cluster determination (Fig. S4†). It should be noted, however, that these parameters can also be readily tuned to suit different user applications; for instance, the seed density factor threshold could be increased for applications where biological outcome is contingent on tightly-packed cell clusters (*e.g.* organoid cultures). In our case, we selected a slightly lower seed density threshold that provided a near-optimal correlation coefficient but also included cells that were more loosely associated with the patterned clusters. Having defined the cell clusters from the Voronoï diagrams, we were able to extract quantitative information regarding the patterned cell array, such as the number, area, barycenter and density of the clusters, as well as the proportion of clustered seeds. Using these metrics, we were able to numerically and statistically compare patterning across different experimental conditions.

As a proof-of-concept demonstration, we used Voronoï tessellation analysis to quantify the clusters formed using different concentrations of myoblasts (1.25 × 10^5^–2 × 10^6^ cells per mL). We observed that using a higher concentration of cells resulted in negligible change in cluster number but rather the formation of larger sized clusters (Fig. 1f and S5A and B†). These profiles were expected, given the conserved total area and number of pressure nodes in the system. Indeed, the capacity to generate uniform and well-defined clusters is a valuable characteristic of acoustic cell patterning and one that has been used to exert control over spheroid culture and organoid tissue engineering.^11^–^13^ In this context, accurate measurement of cluster size could be used to determine size thresholds for the formation of nutrient gradients or the developmental fate of stem cells. However, it should be noted that this analysis provides a measure of cluster area not volume. This is not an issue for many systems: for instance, the clusters we formed using a low concentration of cells predominantly occupied a single layer. On the other hand, when cells are patterned at high concentration, they can occupy multiple layers within an acoustic node and may not contribute to the cluster size measurements made using Voronoï tessellation analysis. Indeed, this effect was evident in the non-linear relationship that we observed between cell concentration and cluster area (Fig. S5C†).

**Fig. 2:**
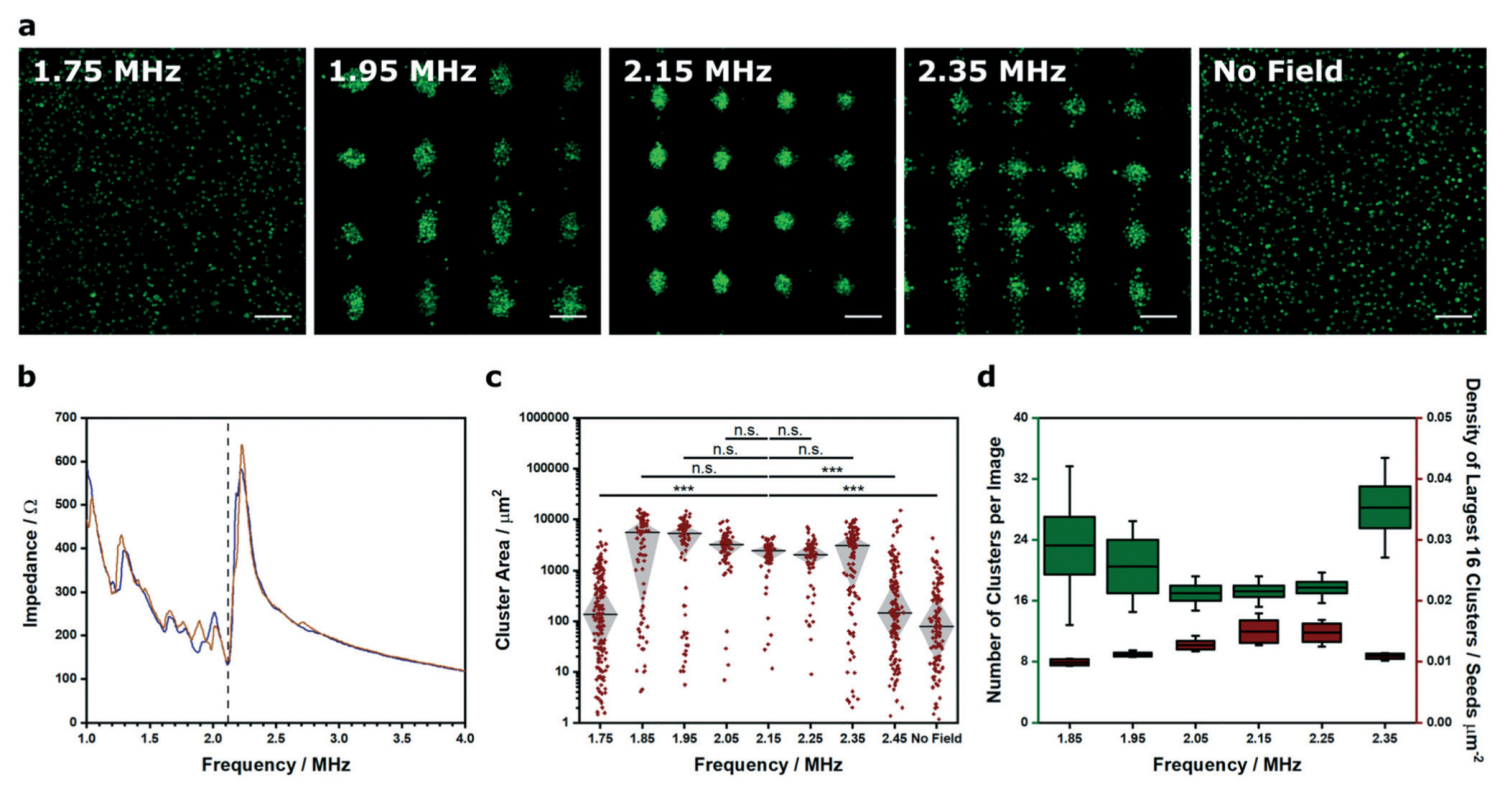
Voronoï tessellation analysis of clusters formed at different ultrasound frequencies. (a) Representative confocal fluorescence micrographs of myoblasts (green) imaged after a 5 min exposure to an acoustic field across a range of ultrasound frequencies. Scale bars = 200 μm. (b) Impedance spectroscopy was used to characterize the frequency response for each piezotransducer pair (blue and orange traces). An impedance minimum corresponding to the material resonance was identified at 2.12 MHz for each pair (black dashed line). (c) Voronoï tessellation was used to quantify the cluster area at different frequencies. Tight clusters were formed around the piezotransducer resonance (2.05–2.25 MHz), while larger, more polydisperse clusters were observed at the shoulders of the resonant frequency (1.85, 1.95, 2.35 MHz). Data was collected from four separate images per group, plotted as median ± interquartile range and statistically treated using a Kruskal–Wallis test with Dunn's correction for multiple comparisons (n.s. is nonsignificant, *** is *p* < 0.001). (d) Voronoï tessellation also revealed a minimum in the number of clusters per image (green box plot, left *y*-axis) at the frequencies close to resonance (2.05–2.25 MHz). This trend mirrored a peak maximum observed in the density of the largest 16 clusters of each image (red box plot, right *y*-axis). Data was collected from four separate images per group, and plotted as the mean, interquartile range and 95% confidence intervals.

We were able to generate Voronoï diagrams from a range of cell structures, including low-frequency clusters (2.1 × 2.1 MHz), high-frequency clusters (6.7 × 6.7 MHz) and linear arrays (2.1 MHz) (Fig. S6†). In these examples, we employed frequencies close to the known primary resonance and first harmonic of the piezotransducer in order to produce steep pressure gradients and well patterned clusters. Indeed, the piezotransducer resonance often dominates the chamber resonance of the device and can be viewed as one of the most critical factors for acoustic cell patterning. To investigate this effect, we captured confocal fluorescence micrographs of myoblasts patterned in cell medium using transducers driven at 15 V_pp_ (corresponding to a mean squared pressure amplitude of 0.0051 (MPa)^2^ for this device) across a range of ultrasound frequencies (1.75–2.45 MHz) (Fig. 2a). This range was selected as it centered on the primary resonance of the piezotransducers (2.12 MHz), which was identified using impedance spectroscopy (Fig. 2b). We then used Voronoï tessellation analysis to quantify the cell cluster area, which revealed a strong association between the ultrasound frequency and the final pattern quality (Fig. 2c). As expected, the frequencies close to the piezotransducer resonance (2.05, 2.15, 2.25 MHz) produced a tight distribution of large clusters, due to effective acoustic patterning. The more distant frequencies (1.75, 2.45 MHz) generated clusters with statistically-significant differences in area distribution to the resonance condition (2.15 MHz) and much more similar to the control with no applied field. Moreover, the frequencies on the shoulder of the piezotransducer resonance (1.85, 1.95, 2.35 MHz) produced clusters of intermediate size distribution, and interestingly, the highest median cluster area of the tested range. This result was attributed to the fact that offresonance patterning produces loosely aggregated cell clusters with a greater size than the tightly-packed clusters formed at resonance. Indeed, we used Voronoï tessellation to calculate the number of clusters per image and the density of the largest 16 clusters across the patterned range (Fig. 2d). This analysis revealed a strong association between the cluster number minimum and the cluster density maximum, which were both centered around 2.15 MHz.

**Fig. 3:**
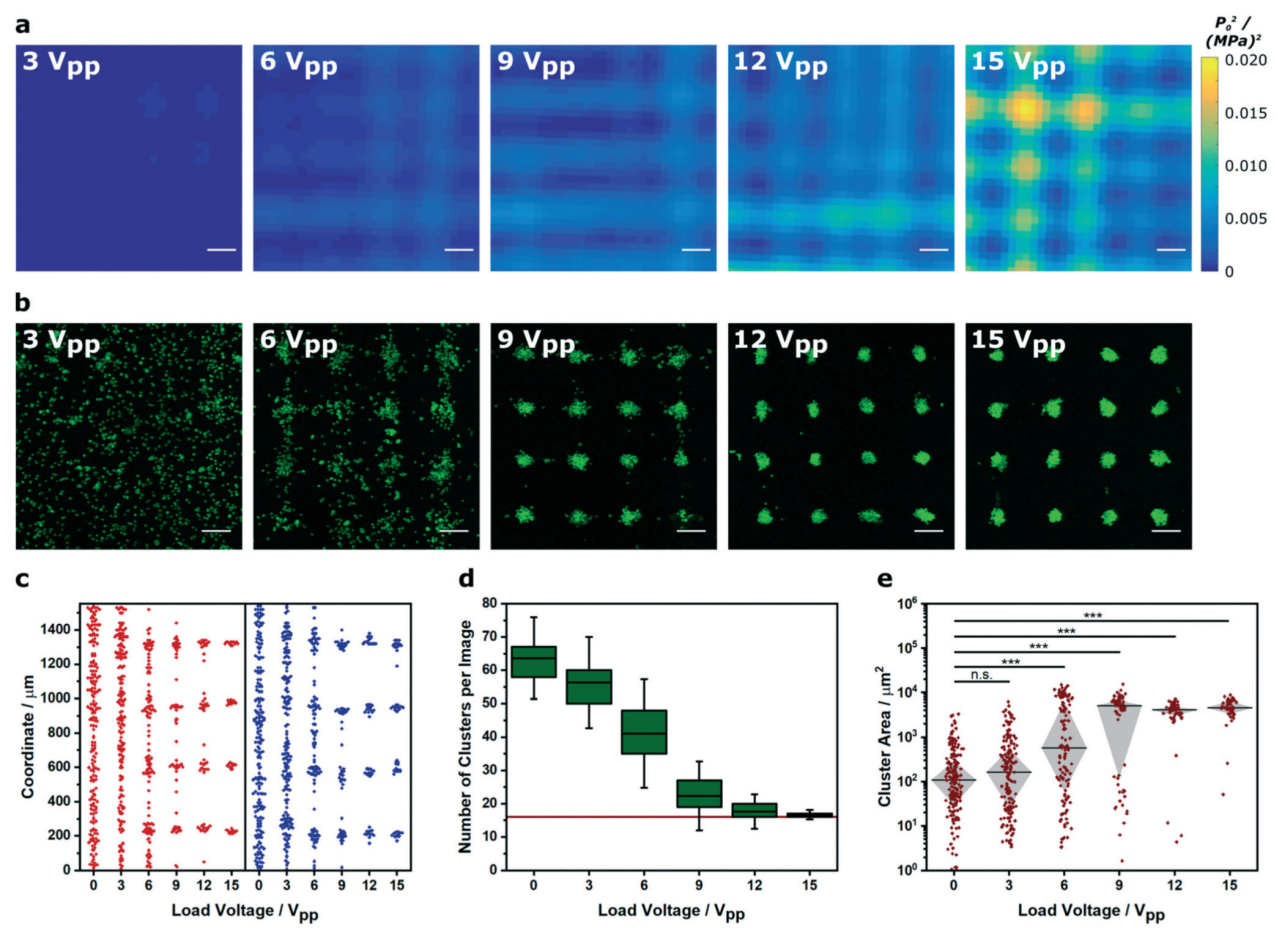
Voronoï tessellation analysis of clusters formed in different pressure amplitude fields. (a) A hydrophone was scanned across the center of an acoustic patterning device to map the mean squared pressure amplitude at different load voltages. Scale bars = 200 μm. For low magnification maps, refer to Fig. S7.† (b) Representative confocal fluorescence micrographs of myoblasts (green) imaged after a 5 min exposure to an acoustic field across the same voltage range. Scale bars = 200 μm. (c) The cluster barycenter coordinates, output from the Voronoï tessellation analysis, were plotted for each ultrasound standing wave pair (*x* = red, *y* = blue). The higher amplitude fields produced clusters with barycenters localized at periodic intervals corresponding to the acoustic nodes of the standing wave. Data was collected from three separate images per group. For a full analysis, refer to Fig. S8 and 9.† (d) Voronoï tessellation was also used to quantify the number of clusters per image. In higher amplitude fields, this value tended towards 16 (red line), which was equivalent to the number of acoustic nodes in each field of view. Data was collected from three separate images per group, and plotted as the mean, interquartile range and 95% confidence intervals. (e) Raising the pressure amplitude also produced an increasingly tight distribution of large cell clusters. Data was collected from three separate images per group, plotted as median ± interquartile range and statistically treated using a Kruskal–Wallis test with Dunn's correction for multiple comparisons (n.s. is nonsignificant, *** is *p* < 0.001).

We next investigated how Voronoï tessellation could be used to characterize myoblasts patterned in solution across a range of pressure amplitudes. First, we used a hydrophone mounted on a motorized stage to map the pressure field generated by a 2.1 × 2.1 MHz ultrasound field at different load voltages (0–15 V_pp_). Unsurprisingly, the devices driven with higher load voltage produced standing waves with higher mean squared pressure amplitude (Fig. 3a and S7†). Importantly, the steeper pressure gradients produced by the higher load voltages appeared to produce more defined cell clusters (Fig. 3b). For this analysis, we identified the barycenter of the clusters detected from the Voronoï tessellation maps and mapped their *x* and *y* coordinates individually, in order to assess the performance of each piezotransducer pair (Fig. 3c). This analysis, which we also performed in two dimensions (Fig. S8†), showed that driving the patterning device at higher load voltage generated a more periodic distribution of barycenter coordinates. Indeed, when the higher load voltage systems were fitted to a tetramodal curve, we were able to measure peak-to-peak separation distances of 0.36 ± 0.02 and 0.36 ± 0.03 mm that were consistent with the theoretical half wavelength separation of the acoustic pressure nodes (0.35 mm, using an ultrasound frequency of 2.1 MHz and a speed of sound in water of 1482 m s^−1^) (Fig. S9†).^41^ This geometric evaluation was consistent with numerical and dimensional analysis. Each micrograph encompassed a 4 × 4 array of pressure nodes, so in theory, a perfectly patterned system would comprise 16 uniformlysized clusters. Voronoï tessellation analysis revealed the 15 V_pp_/0.0051 (MPa)^2^ system to have a small number of clusters (*N* = 17 ± 1) with a relatively large median cluster size (*Ã* = 4.6 ± 0.3 × 10^3^ μm^2^). When we reduced the load voltage, we observed a large increase in cluster number and a concomitant decrease in cluster area (Fig. 3d and e). These observations are consistent with theory; the acoustic radiation force experienced by a cell is proportional to the mean squared pressure (*F* α〈|*p*_0_|^2^〉). Although a radiation force will be exerted in all field-exposed systems, this analysis showed that the load voltage must exceed a certain threshold limit (6 V_pp_/0.0017 (MPa)^2^) in order to produce pressure gradients capable of generating detectable cell clusters.

**Fig. 4:**
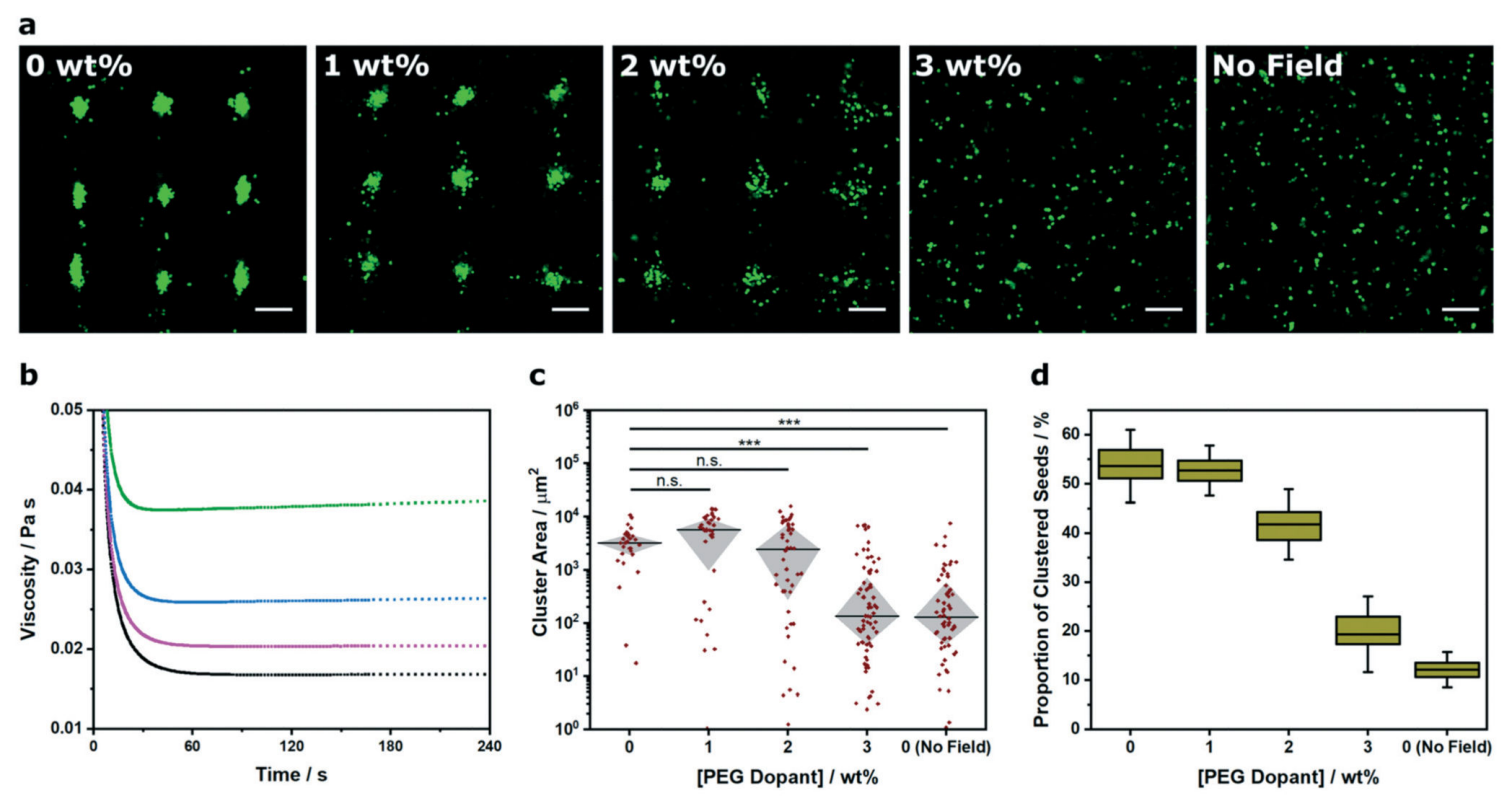
Voronoï tessellation analysis of clusters formed in different viscosity systems. (a) Representative confocal fluorescence micrographs of myoblasts (green) acoustically patterned within PEG norbornene hydrogels containing a concentration range of high molecular weight PEG dopant (0–3% w/v). The hydrogels were photocrosslinked after 5 min of patterning, swollen overnight in PBS and then imaged. An undoped control was included without any applied field. Scale bars = 200 μm. (b) Rheological creep tests at 2 Pa stress were used to characterize the viscosity of the PEG norbornene precursor solution with a PEG dopant concentration of 0% (black), 1% (magenta), 2% (blue) and 3% (green). (c) The cluster area profile was measured for each dopant concentration to provide a quantitative measure of pattern formation across the viscosity range. Data was collected from three separate images per group, plotted as median ± interquartile range and statistically treated using a Kruskal–Wallis test with Dunn's correction for multiple comparisons (n.s. is nonsignificant, *** is *p* < 0.001). (d) Voronoï tessellation was also used to measure the proportion of total seeds that were detected within a cluster. Data was collected from three separate images per group and plotted as mean, interquartile range and 95% confidence intervals.

Recently there has been interest in moving away from solution-based arrays towards approaches that use biomaterial systems to encapsulate and preserve acousticallypatterned cell arrays (*e.g.* hydrogels of poly(ethylene glycol) norbornene,^15^ Matrigel,^15^ fibrin,^16^,^17^ alginate,^30^ agar/agarose,^15^,^30^ collagen,^15^,^19^,^20^,^42^ gelatin methacryloyl^15^,^18^). In these examples, cells are acoustically patterned in a liquid hydrogel precursor before or during a triggered crosslinking process (*e.g.* enzymatic, thermal, pH, ultraviolet irradiation). The chemical and physical properties of the biomaterial components, the total weight percentage and any pre-gelation crosslinking will dictate the rheological properties of the liquid precursor, and thus the degree of constraint placed upon the acoustic patterning process. Accordingly, we used Voronoï tessellation to assess the relationship between viscosity and cluster formation (Fig. 4a). For this study, we used a two-component hydrogel system, namely a photo-crosslinkable 8-arm PEG norbornene hydrogel precursor that was systematically doped with high molecular weight PEG (0–3% w/v). We used rheological creep tests to measure a respective viscosity of 16.7 ± 0.1, 20.7 ± 0.2, 26.2 ± 0.2 and 38.8 ± 0.2 mPa s for the systems containing 0, 1, 2 and 3% (w/v) PEG dopant (Fig. 4b). We used a 2.1 × 2.1 MHz field to acoustically pattern myoblasts within these four hydrogel precursors, followed by a 2 min exposure to ultraviolet light, in order to immobilize the arrays in a self-supporting hydrogel (see Experimental for more details). The center of the hydrogel contained a uniform array of clusters (Fig. S10†), which we imaged and analyzed using Voronoï tessellation analysis. As expected, the cluster size distribution was impacted by increasing the viscosity, with 3% dopant producing a cluster area profile with statistically-significant difference to the undoped system (Fig. 4c). Moreover, the proportion of clustered seeds (*Z*) was markedly decreased in the 2% (*Z* = 42 ± 3%) and 3% (*Z* = 19 ± 3%) systems compared to the undoped hydrogel (*Z* = 54 ± 3%) (Fig. 4d). These results highlight the implications for acoustic patterning applications that require high viscosity hydrogels. More generally, this route offers a means to quantifiably determine the viscosity limits for acoustic patterning in different biomaterial systems.

**Fig. 5:**
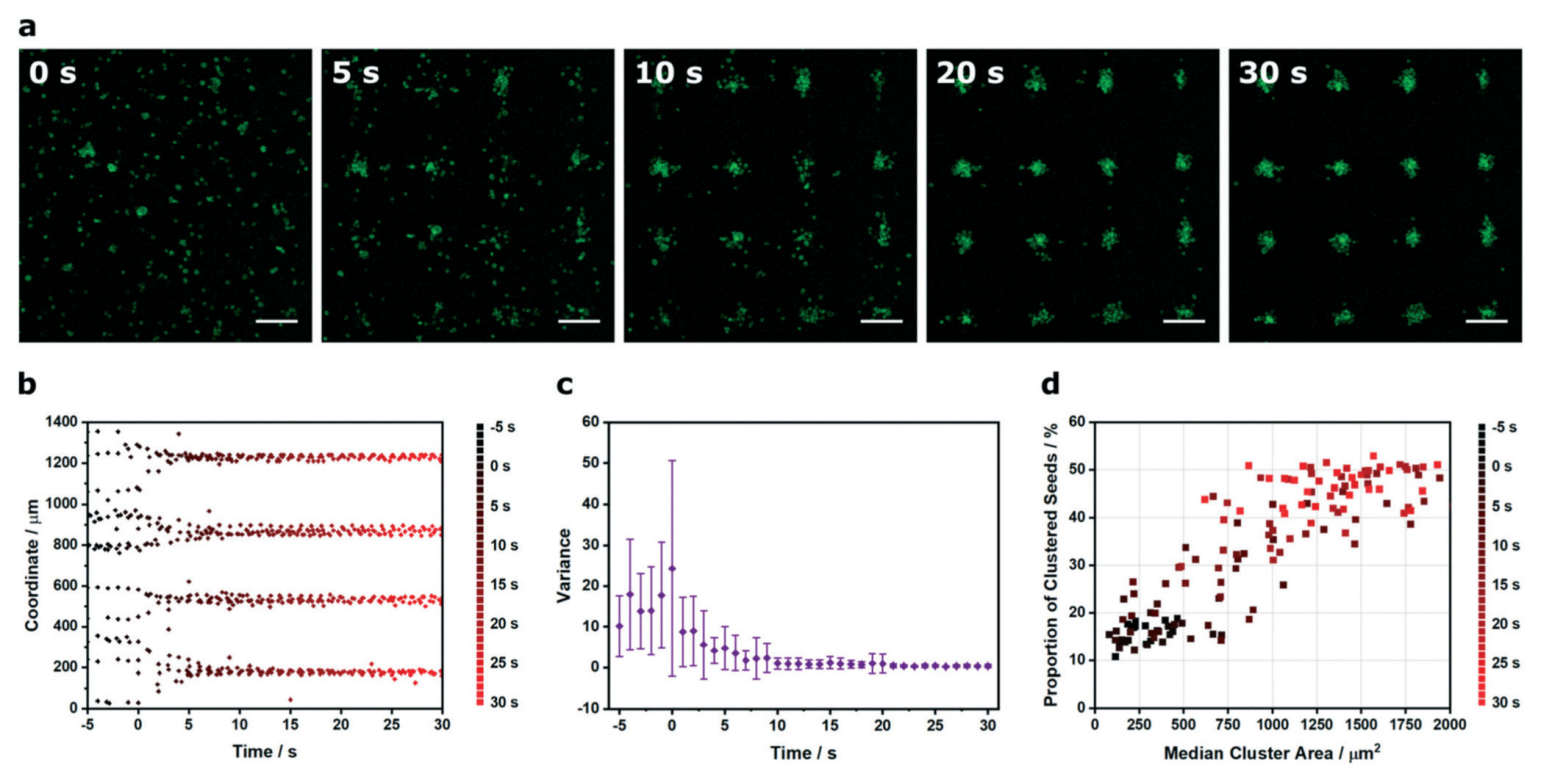
Spatiotemporal Voronoï tessellation analysis of the acoustic cell patterning process. (a) Time-lapse confocal fluorescence micrographs of myoblasts (green) in culture medium, imaged at intervals during acoustic cell patterning. Scale bars = 200 μm. (b) The *x* and *y* coordinates of the cluster barycenters were plotted as a function of time. Data was collected from four separate videos of patterning, here, one representative x coordinate dataset is shown. For the full coordinate analysis, refer to Fig. S11.† (c) The cluster barycenter analysis was used to define histogram profiles at 1 s intervals, which were fitted to a tetramodal Gaussian distribution mixture based on the expectation maximization algorithm. The variance on the four identified peaks was relatively high in the initial unpatterned system but decreased as the acoustic patterning generated periodic cell arrays. Data plotted as mean ± standard deviation from tetramodal fits of four separate videos of patterning, with only the *x* coordinate data shown. For all data fitting and the y coordinate variance analysis, refer to Fig. S12 and 13.† (d) This process could also be visualized by describing the proportion of clustered seeds as a function of median cluster area. A clear transition could be observed between two distinct regions: the unpatterned cell suspension with small clusters and a low proportion of clustered seeds (*t* < 3 s) and the patterned arrays with large clusters and a high proportion of clustered seeds (*t* > 13 s).

The results described thus far demonstrate the capacity of Voronoï tessellation for quantifying cluster formation at single timepoints during the acoustic cell patterning process. In practice, however, the cluster definition improves over time as the acquired potential energy of the cells is converted into kinetic energy and the cells are translated towards the acoustic pressure nodes. Here, we used timelapse microscopy and Voronoï tessellation to investigate the dynamics of the acoustic patterning process. Specifically, we employed *in situ* confocal fluorescence microscopy to capture the patterning of myoblast clusters formed using a 2.1 × 2.1 MHz ultrasound field (Fig. 5a and video S1†). We tracked the *x* and *y* barycenter coordinates of the clusters, both prior to field exposure (−5 to 0 s) and during patterning (0 to 30 s) (Fig. 5b and S11†). This revealed a transition from a broadly uniform distribution to a periodic profile comprising four distinct peaks. In order to quantify this system, the profiles were fitted to a tetramodal Gaussian distribution mixture based on the expectation maximization algorithm^43^ and the variance of the peaks expressed as a function of time (Fig. 5c and S12 and 13†). This revealed a rapid decrease in variance, as the cell patterning transitioned the system from relatively uniform distributions into periodic arrays fitted with narrow Gaussian distribution curves. In addition to this coordinate analysis, the cluster area and proportion of clustered seeds proved to be particularly insightful metrics for characterizing the patterning process (Fig. 5d). For the five seconds prior to field exposure, the system was predominantly composed of small clusters (*Ã* < 5 × 10^2^ μm^2^) and a low proportion of clustered seeds (*Z* < 20%). Initiating the patterning process produced rapid increases in both cluster area and proportion of clustered seeds to produce a final system that exhibited a stable and consistent profile after 20 s of patterning (*Ã* > 6 × 10^2^ μm^2^, *Z* > 35%). This analysis clearly demonstrates how Voronoï tessellation can be used for spatiotemporal quantification and kinetic evaluation of acoustic cell patterning.

**Fig. 6:**
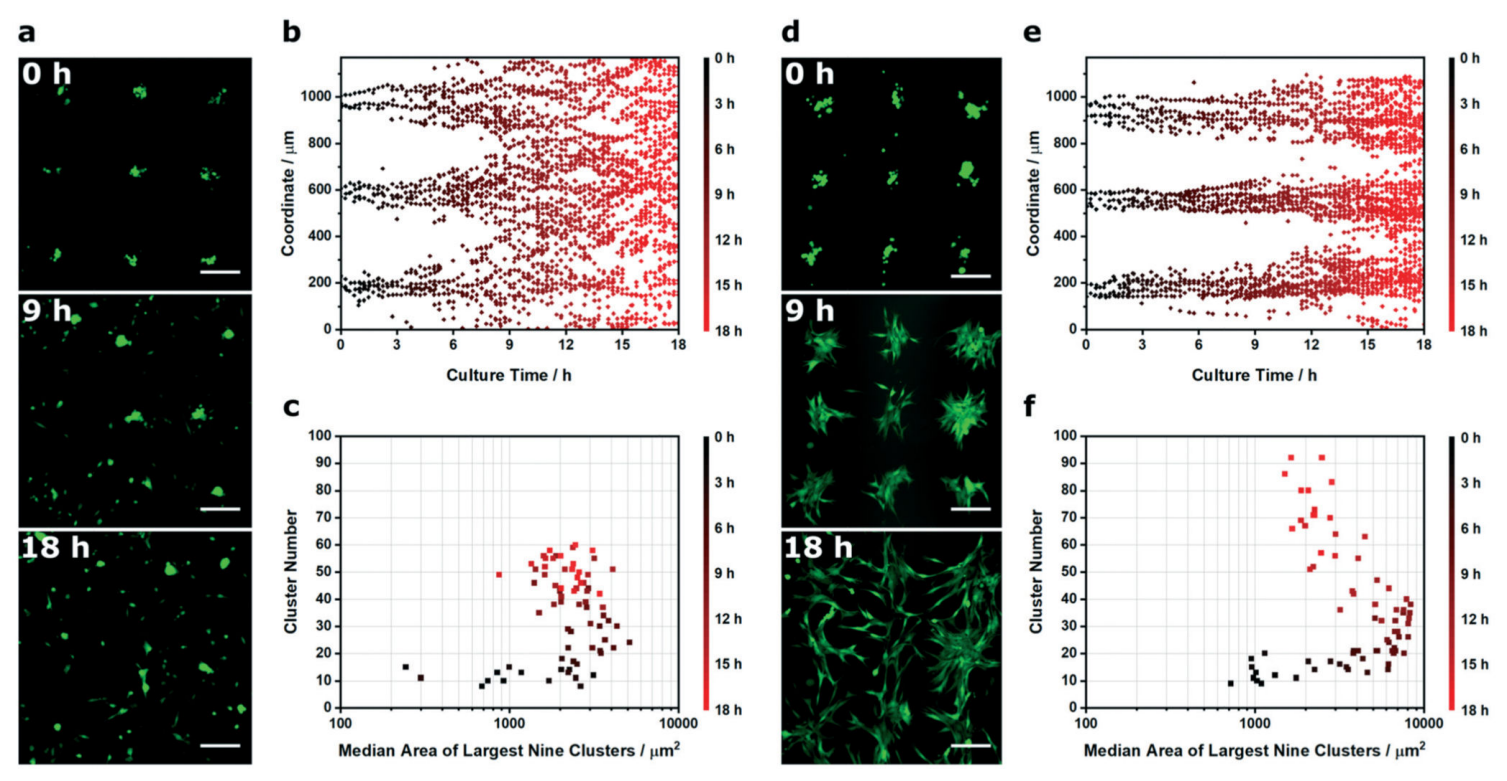
Spatiotemporal Voronoï tessellation analysis of patterned cell migration. (a) Time-lapse wide field micrographs of acoustically-patterned myoblasts (green) adhered to a laminin-coated glass substrate, imaged during culture in expansion medium. (b) Spatiotemporal Voronoï tessellation was used to identify clusters at each time point, with the *x* and *y* coordinates of the cluster barycenters plotted over time. Here, only the x coordinate dataset is shown. (c) Voronoï tessellation was also used to track the median area of the largest nine clusters (those corresponding to the clusters formed at the acoustic nodes) and the number of clusters per image. (d) Matrigel was used to slow the migration of the myoblasts from the original pattern, with an identical spatiotemporal analysis used to define the change in (e) cluster barycenter coordinates, (f) the median area of the largest nine clusters and the number of clusters per image. In each case, the Voronoï tessellation analysis was used to track a single timelapse video. All scale bars = 200 μm.

We next applied Voronoï tessellation to characterize spatiotemporal changes that occur after acoustic cell patterning. For this study, we used a common experimental approach of patterning cells onto a substrate and then removing the acoustic field once the cells have adhered.^24^,^25^,^27^,^33^ Without the field, there are no external forces acting to constrain the cells to their patterned configuration.^25^ As a result, there is almost always some degree of pattern loss over time, due to normal cell processes such as membrane spreading, migration and proliferation.^24^,^25^,^27^ Generally, this effect is greater than for cells grown on surfaces engineered with topographic, mechanical or chemical cues, which are able to exert long-term effects during culture.^44^–^46^ In some cases, pattern loss is slow and does not hinder the final application: for instance, Gesellchen *et al.* showed that Schwann cells largely retained their patterned configuration on glass for at least 24 h, and could be used to guide neurite outgrowth from a dorsal root ganglion for a further four days.^27^ However, pattern loss will depend on factors such as the cell type and environment: for instance, Li *et al.* showed that linear arrays of HeLa cells were reasonably well preserved after 24 h in monoculture, but over the same time period, cocultured HeLa cells and endothelial cells each lost their patterned configuration.^24^ To the best of our knowledge, the only reported analysis of acoustically-patterned cultures involved the tracking of individual cells^24^ and individual clusters.^25^ Accordingly, we sought to characterize the population-wide pattern loss using live-cell microscopy of C2C12 myoblasts expressing cytosolic GFP. Specifically, we generated uniform clusters of adherent myoblasts on the surface of laminin-coated glass, and then imaged the cells over 18 h of culture using timelapse wide field microscopy (Fig. 6a).

As before, we used Voronoï tessellation to measure the distribution in the cluster barycenter coordinates over time. This provided an effective means of visualizing the loss of pattern fidelity, with the clusters spreading out from the acoustic nodes to fully occupy the coordinate space (Fig. 6b). We also used Voronoï tessellation to monitor the median area of the nine largest clusters (*Ã*) and the total number of clusters over time (*N*), which provided interesting insights into the dynamic changes occurring over the culture period (Fig. 6c). During the first hour of culture, in the early stages of cell-substrate adhesion, the initial patterning profile was well maintained (*Ã* < 1.0 × 10^3^ μm^2^, *N* < 15 at *t* < 1 h). After this initial lag period, the clusters remained largely intact but underwent membrane spreading; this was characterized by an unchanged cluster number but an increase in cluster size (*Ã* = 2.2 × 10^3^ μm^2^, *N* = 13 at *t* = 3.5 h). The next transition saw a steady increase in cluster number, due to the outward migration of myoblasts from the patterned clusters (*Ã* = 2.5 × 10^3^ μm^2^, *N* = 60 at *t* = 18 h). This rapid deterioration in pattern quality affords an exceptionally small window of opportunity to study biological processes, a factor that has limited acoustically-patterned culture systems to a small number of studies with short term outcomes.^24^,^27^ We hypothesized that we could extend this time frame by restricting migration with a constraining matrix. Indeed, by layering Matrigel on the surface of the adherent myoblasts we were able to slow cell migration and retain the acoustically-formed clusters over a greater period of time (Fig. 6d and e). Interestingly, the eventual loss of definition in the Matrigel system appeared to be dominated by cell proliferation, as opposed to the outward migration seen in the uncoated clusters. This was reflected in the Voronoï tessellation metrics; the coated system produced appreciably larger clusters during the intermediate stages (*Ã* = 8.3 × 10^3^ μm^2^ at *t* = 10 h) than in the uncoated system (*Ã* = 2.8 × 10^3^ μm^2^ at *t* = 10 h) (Fig. 6f). This migration analysis provides a further demonstration of how Voronoï tessellation can be applied to quantitatively characterize spatiotemporal processes in acoustically-patterned cell cultures.

Finally, we sought to demonstrate that Voronoï tessellation analysis can be applied to acoustically-patterned cells that were not labelled with any fluorescent marker. We generated 5% (w/v) PEG norbornene hydrogels patterned with unlabelled myoblasts using a 2.1 × 2.1 MHz ultrasound field, as described previously. We removed the hydrogel from the device and imaged the clusters using bright field, differential interference contrast and phase contrast microscopy (Fig. S14†). We were able to analyze these images using Voronoï tessellation, with little difference observed between the identified clusters.

## Conclusions

Acoustic cell patterning is rapidly emerging as an important biotechnological platform for guiding cell organization in culture and tissue engineering. While patterning capabilities are rapidly advancing and applications are broadening in exciting new directions, there is a conspicuous absence of quality control checks and quantitative characterisation. Here, we have employed a sophisticated Voronoï tessellation protocol to identify and quantify acoustically-formed cell clusters. Specifically, we have used this technique to extract quantitative information (cluster density, number, area, barycenter, and proportion of clustered seeds) to assess the quality of acoustic patterning under different experimental conditions (pressure amplitude, ultrasound frequency, cell concentration, solution viscosity). Moreover, we have shown that spatiotemporal analysis can be used to quantify the cell patterning process and the post-patterning migration of cells. This analysis is simple, unbiased and highly informative, and we anticipate several areas in which it could be used to aid the acoustic patterning process. As Voronoï tessellation enables numerical and statistical comparison of cell patterning, it can be used to test empirical limits and explore the parameter space of different experimental conditions. In this mode, Voronoï tessellation could be used to determine geometrical thresholds in different biological systems, for instance, to identify the cluster size regimes required for different developmental pathways in acoustically-formed spheroid cultures. Practically, Voronoï tessellation could also be used to provide important quality control checks by quantifying the variation between different ultrasound devices and piezotransducer pairs, as well as their deterioration over time. Taken together, this information may then be used to predict the pattern features of cell/biomaterial systems that are yet to be tested. These varied applications of Voronoï tessellation provide a systematic framework for acoustic cell patterning and will enable a more robust and quantitative approach towards this rapidly developing biotechnology. Moreover, the principles outlined in this report should be readily applied to naturally-formed colonies or clusters,^47^,^48^ as well as organized structures formed using other technologies, such as dielectrophoretic patterning,^49^ material-based cues,^50^ or bioprinted cell systems.^4^

## Supplementary Material

† Electronic supplementary information (ESI) available. See DOI: 10.1039/c8lc01108g

Supplementary Information
