# Supplementary material for: Spatiotemporal quantification of acoustic cell patterning using Voronoï tessellation†
Source: Lab Chip. Author manuscript; Available in PMC 2019 Feb 22. (PMC6386121; doi:10.1039/c8lc01108g)
Supplement: Supplementary Information [file NIHMS81674-supplement-Supplementary_Information.pdf]

Supplementary Information

Spatiotemporal Quantification of Acoustic Cell Patterning using Voronoï Tessellation

James PK Armstrong,[1] Stephanie A Maynard,[1] Isaac J Pence,[1] Amanda C Franklin,[2] Bruce W Drinkwater[2] & Molly M Stevens[1*]

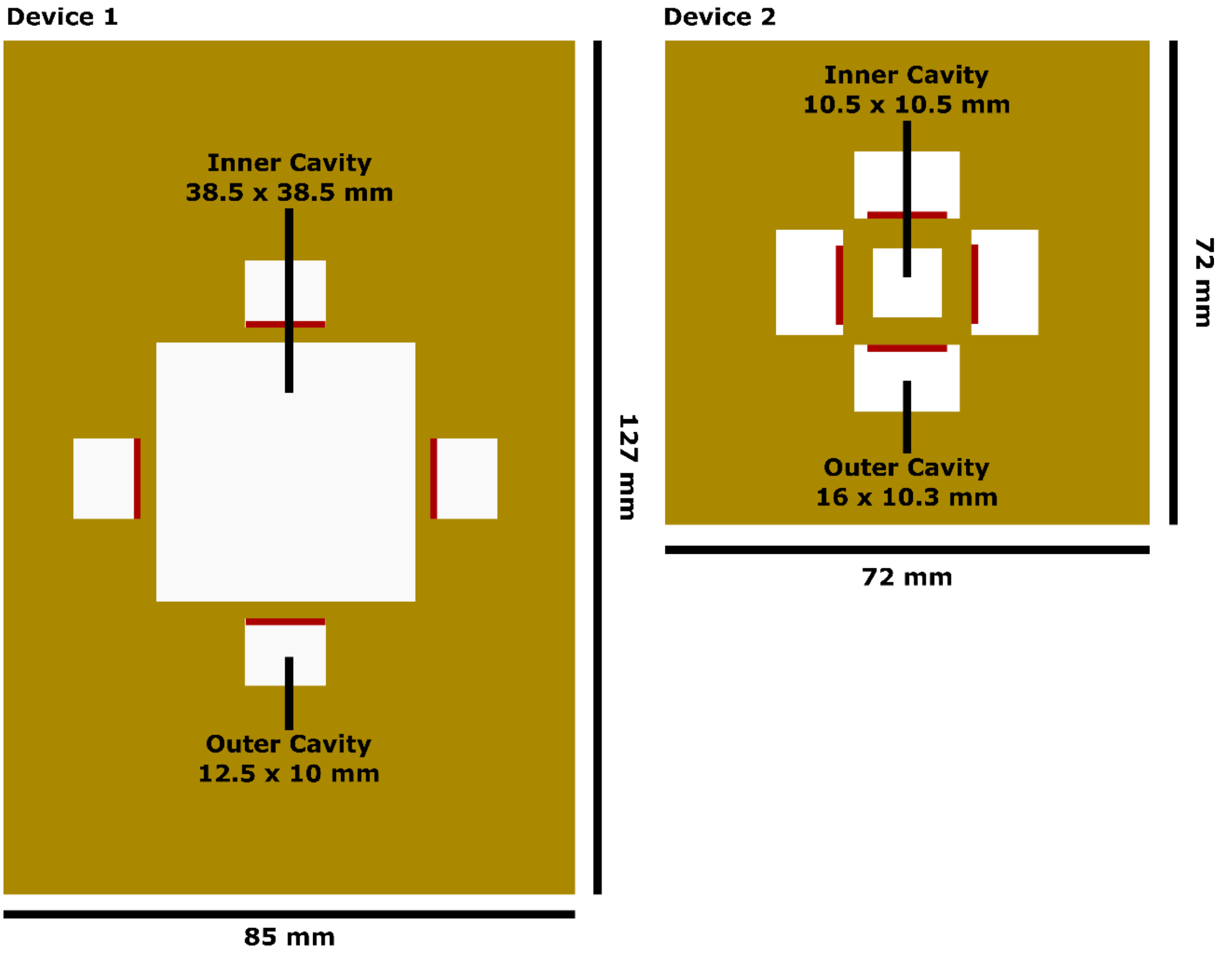

Supplementary Figure 1. Design of the acoustic patterning devices. An acrylic base plate was machined with five cavities. The inner cavity was used to contain the cell suspension and the four outer cavities were used to house affixed piezotransducers (red).

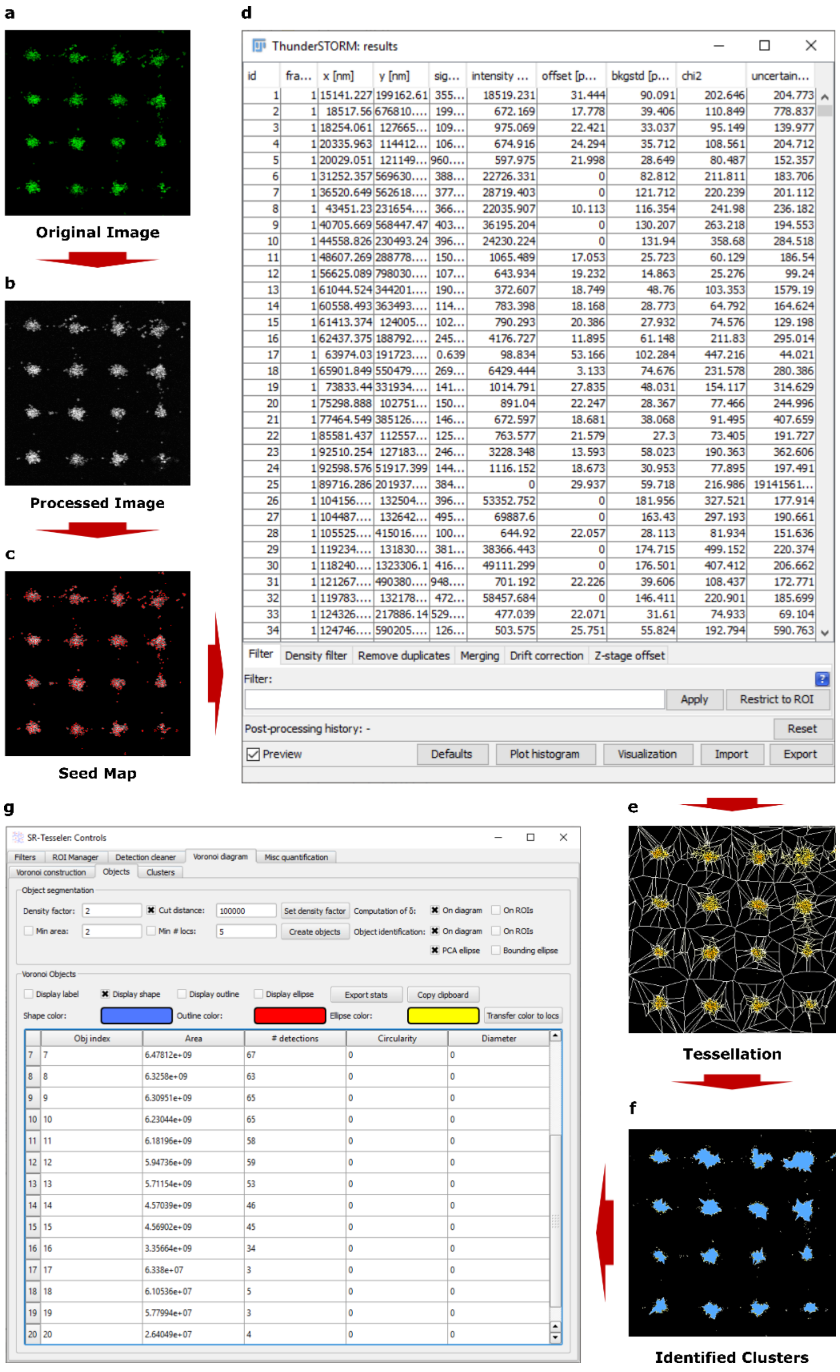

Supplementary Figure 2. Supplementary Figure 2. Process chart for Voronoï tessellation analysis. (a) Original images of acoustically-patterned cells. (b) FIJI was used to convert images into 8-bit format and, if necessary, raise the minimum brightness level to remove any background noise. (c-d) ThunderSTORM (https://github.com/zitmen/thunderstorm) was used to identify seeds and export their co-ordinates as a .csv file. (e) SR-Tesseler (http://www.iins.u-bordeaux.fr/team-sibarita-SR-Tesseler) was used to open this file and generate Voronoï tessellation maps, (f-g) and then identify clusters based on density and cut distance. Cluster information was exported and used in any further analysis.

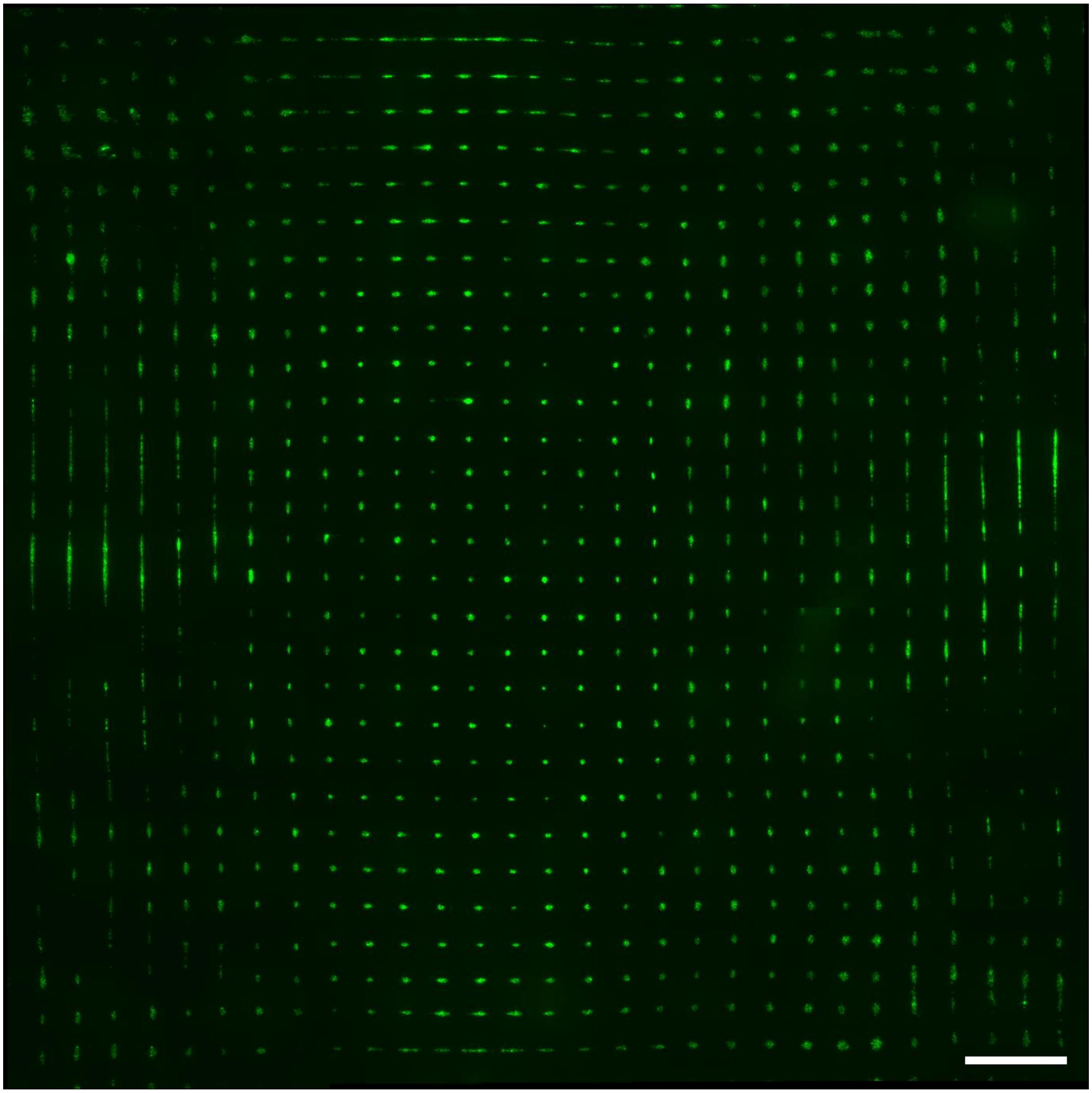

Supplementary Figure 3. Low magnification image of acoustically patterned myoblasts. Representative wide field fluorescence microscope image of myoblasts (green) acoustically patterned in solution using device #1 (2.1 x 2.1 MHz / 0.0051 $(MPa)^2$). A large array of uniform clusters was observed at the centre of the dish: the intersection between the two ultrasound standing waves. Scale bar = 1 mm.

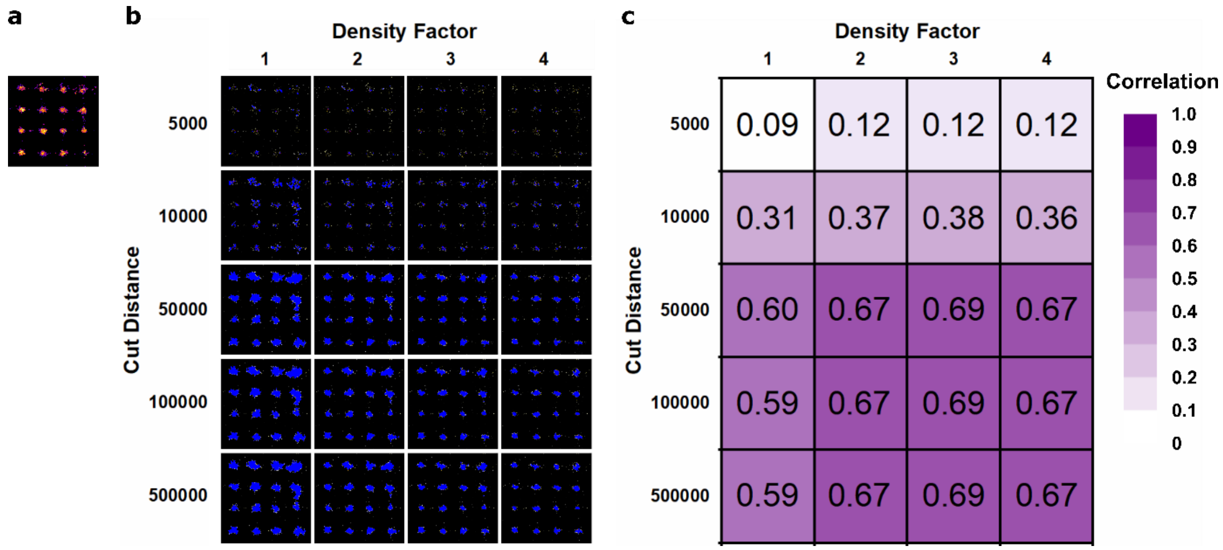

Supplementary Figure 4. Optimizing thresholds for cluster analysis. (a) A representative example of an acoustically patterned cell array and (b) the clusters generated by varying the seed density factor and cut distance. (c) Following bicubic interpolation to downscale the original micrograph to match the dimensions for the generated tessellation maps, a 2D cross correlation function was used to compare the identified cluster pattern with the original image and generate a correlation factor between 0 (no correlation) and 1 (perfectly correlated). This provided an unbiased means of identifying the optimum threshold parameters (seed density factor = 3, cut distance > 50000) with maximum correlation (0.69). However, we used parameters that gave a slightly lower correlation (0.67 using seed density factor 2, cut distance = 100000) to analyse patterning in media and hydrogels. This was chosen on the basis that a lower seed density factor included loosely-associated, clustered cells along with cells tightly packed at the acoustic nodes.

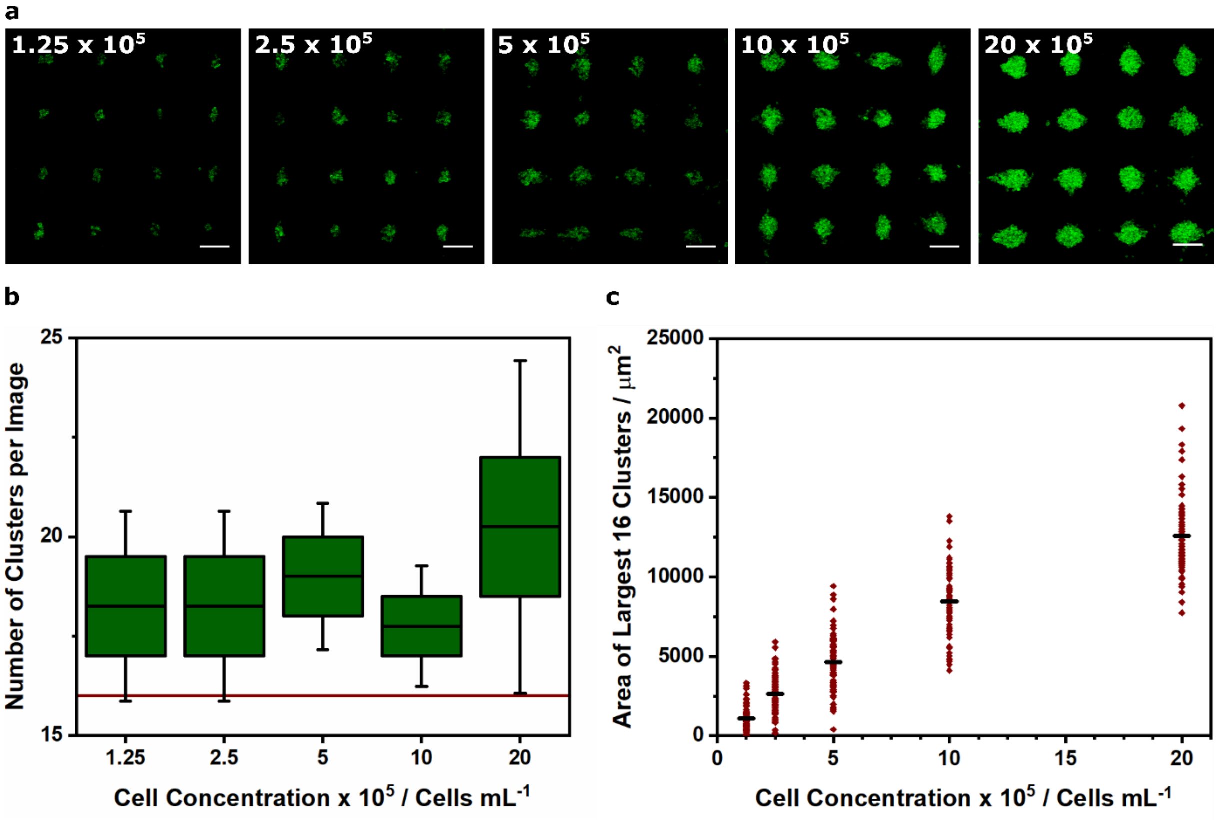

Supplementary Figure 5. Voronoï tessellation analysis of clusters formed at different cell concentrations. (a) Representative confocal fluorescence micrographs of myoblasts (green) imaged after a 5 min exposure to an acoustic field across a range of cell concentrations. Scale bars = 200 µm. (b) Voronoï tessellation was used to quantify the number of clusters per mage. The red line indicates the theoretical number of acoustic nodes present in each image. Data was collected from four separate images per group, plotted as the mean, interquartile range and 95% confidence intervals. (c) Voronoï tessellation was also used to calculate the area of the largest 16 clusters per image (red markers). The mean cluster area (black markers) showed a non-linear response to increasing cell concentration. Data was collected from four separate images per group.

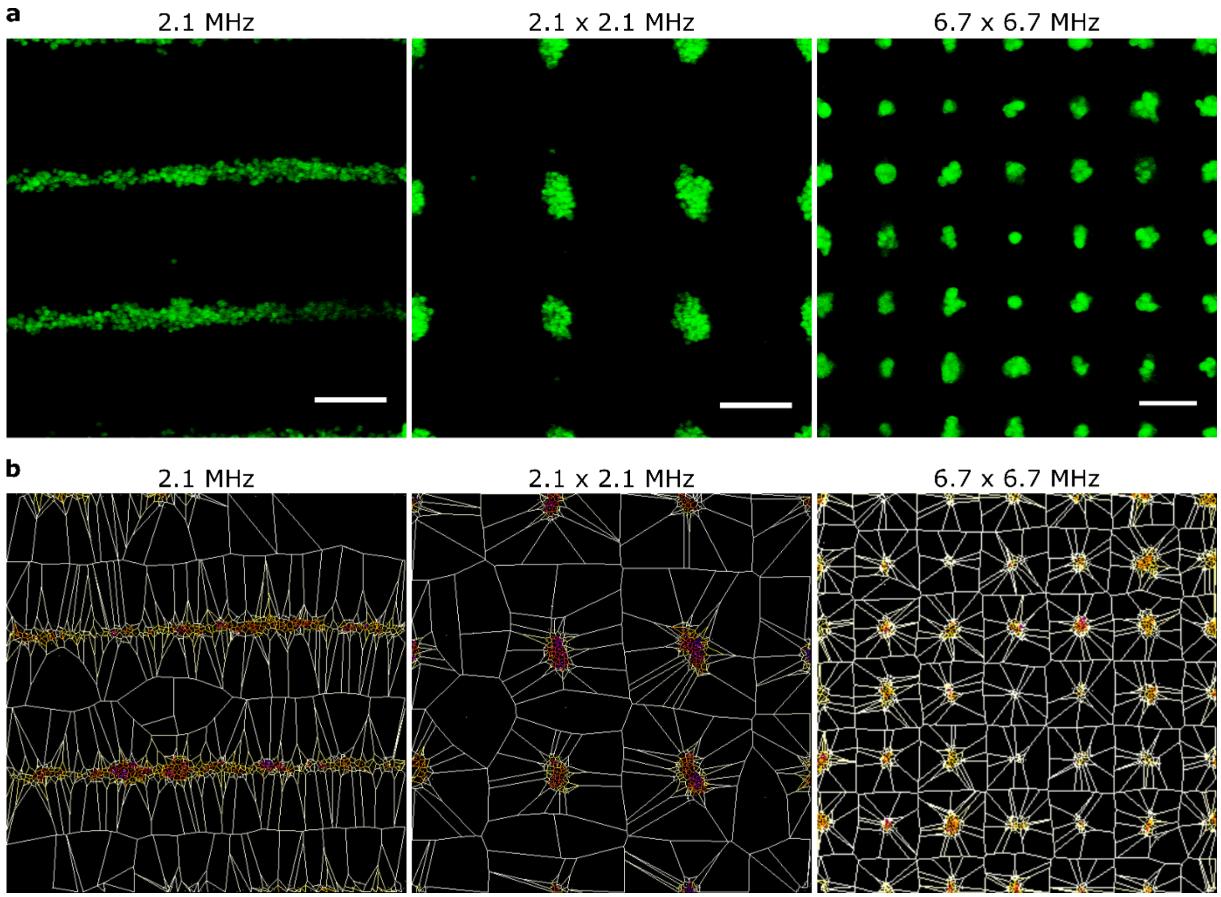

Supplementary Figure 6. Voronoï tessellations of different cell arrays. (a) Confocal fluorescence micrographs and (b) corresponding Voronoï tessellation maps of linear arrays (2.1 MHz), low-frequency clusters (2.1 x 2.1 MHz) and high-frequency clusters (6.7 x 6.7 MHz). Scale bars = 200 µm for the low frequency patterning and 100 µm for the high frequency patterning.

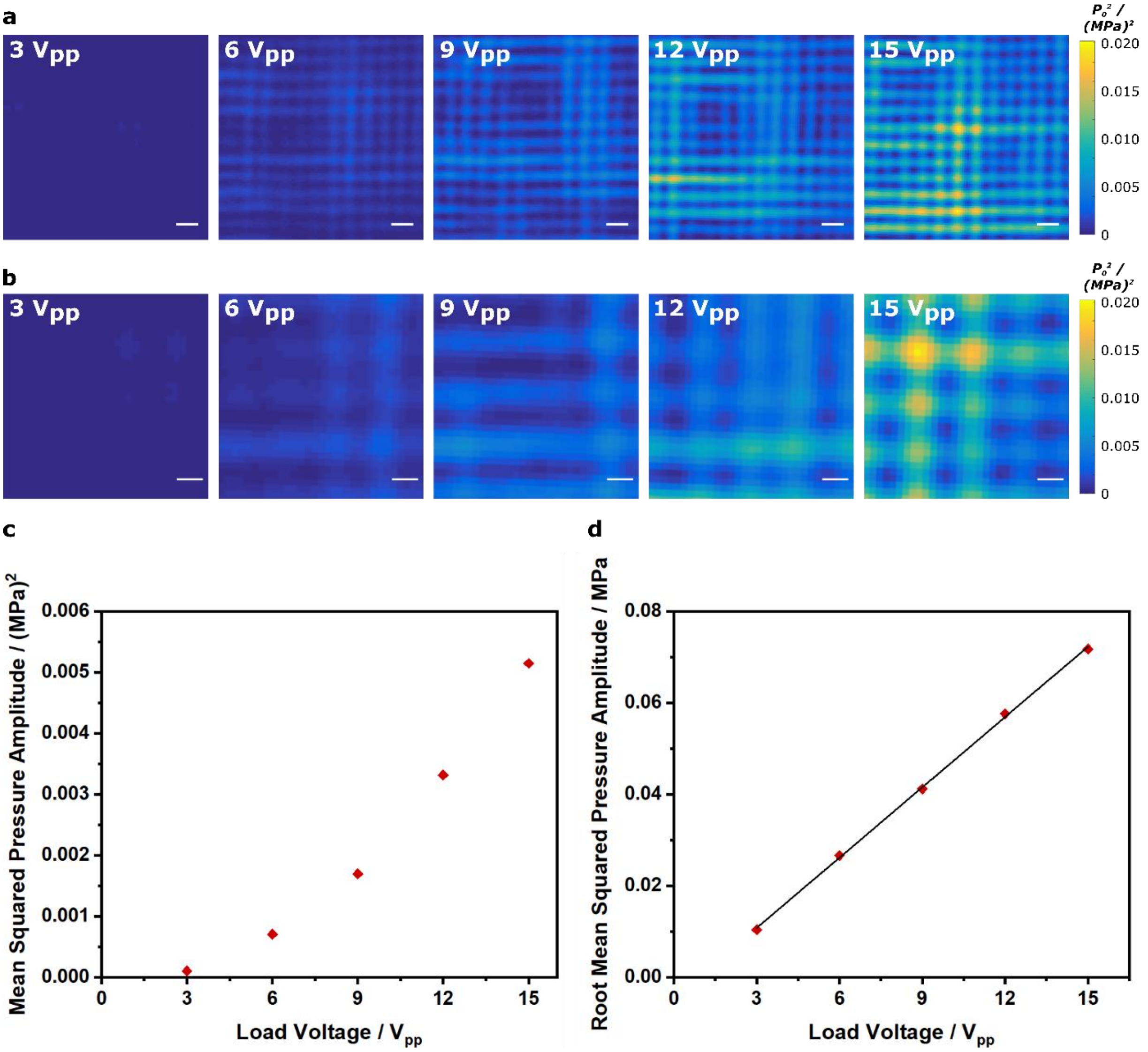

Supplementary Figure 7. Pressure amplitude mapping. (a) A hydrophone was scanned across the centre of an acoustic patterning device to map the mean squared pressure amplitude at different load voltages. Scale bars = 500 µm. (b) Cropped images taken from the same central region of each pressure map. In the higher load voltage systems, 16 pressure nodes can be identified. Scale bars = 200 µm. (c) Driving the device at increased load voltage produced an increase in the mean squared pressure (red markers). Data shown is the mean squared pressure amplitude averaged across a single pressure map for each load voltage. (d) Plotting the root mean squared pressure amplitude (red markers) confirmed that the pressure had linear dependence upon load voltage (black line, R^2 = 0.99973). Data shown is the root mean squared pressure amplitude averaged across a single pressure map for each load voltage.

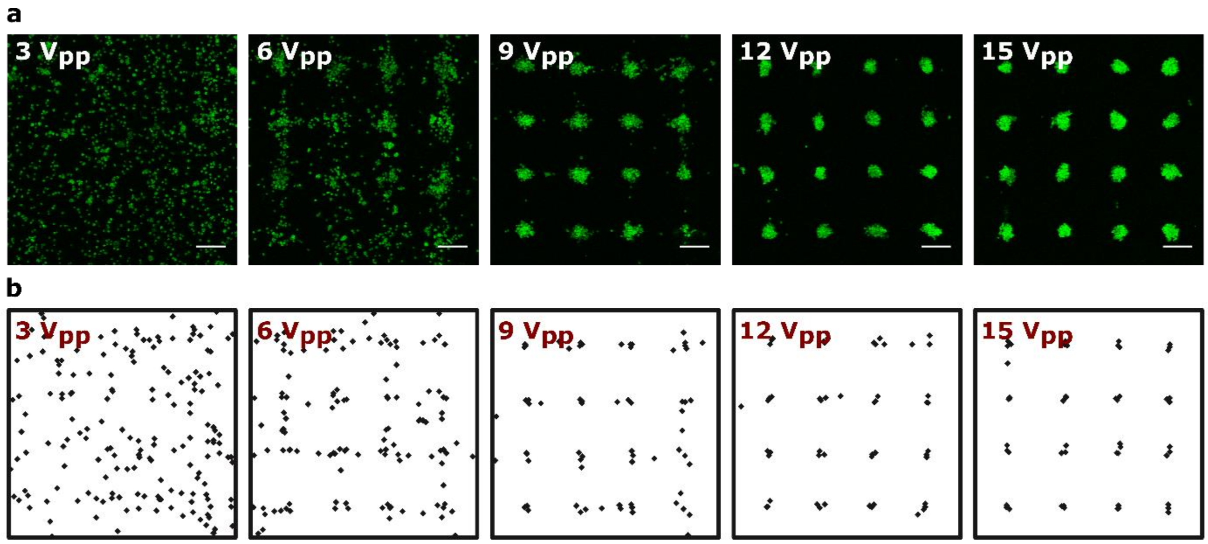

Supplementary Figure 8. Two-dimensional analysis of barycentre coordinates. (a) Confocal fluorescence microscopy images were captured for myoblasts acoustically patterned at different load voltages. (b) Voronoï tessellation analysis enabled the measurement of cluster barycentre coordinates (black markers). At the higher load voltages (> 9 V_{pp}), the cluster barycentres are clearly localized in a periodic formation consistent with patterning at the acoustic pressure nodes. Note that the barycentre coordinates are collated from three separate images per group, while only one representative micrograph is shown.

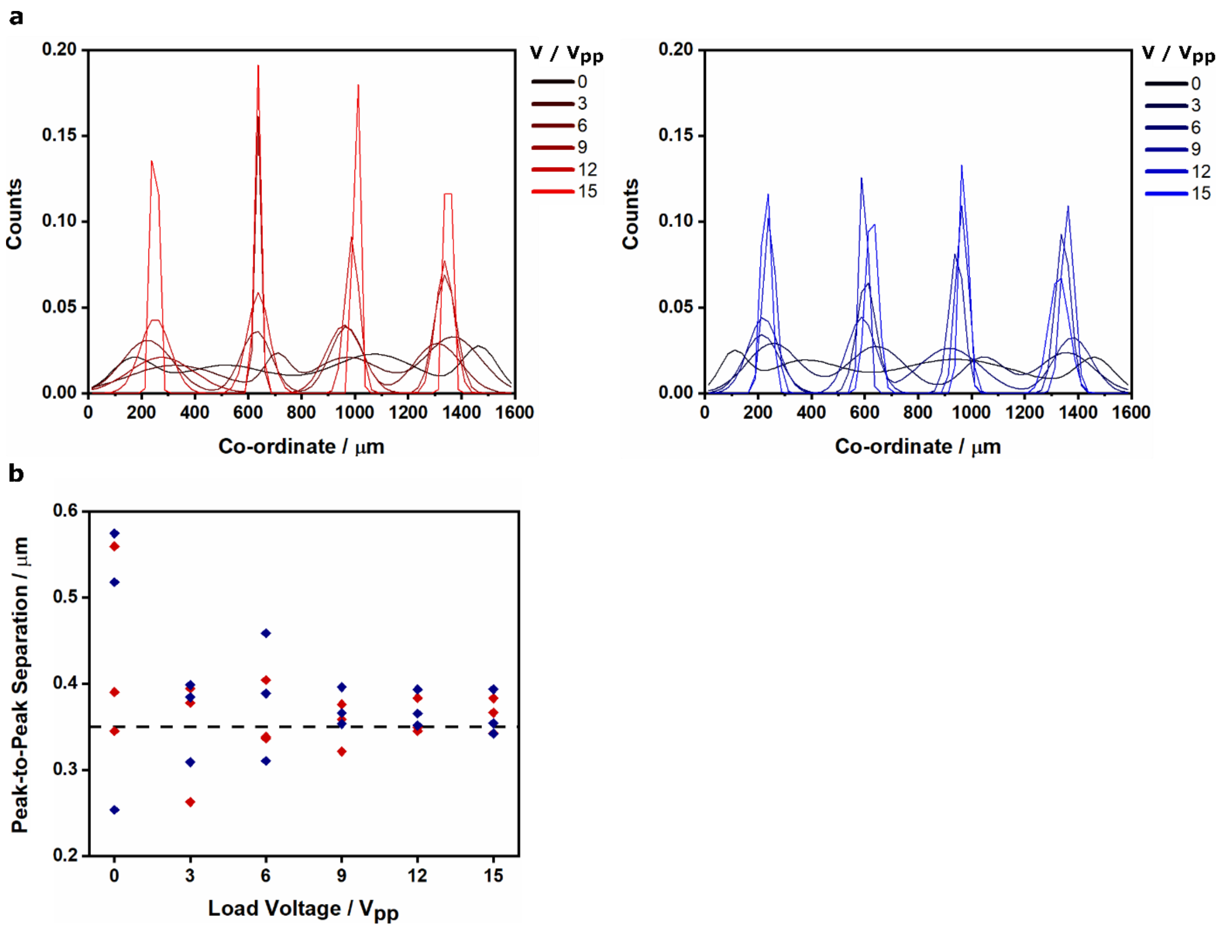

Supplementary Figure 9. Fitting of cluster barycentre distribution. (a) Tetramodal Gaussian distribution mixture analysis curves based on the Expectation Maximization algorithm of the cluster barycentre histograms for different load voltages (See Figure 3C). This analysis showed a transition from uniform distribution at low load voltage to a defined periodic profile at high load voltage, for both the x coordinate (red) and y coordinate (blue) fits. (b) The peak-to-peak separation was calculated from the tetramodal fits and plotted as a function of load voltage. Driving the piezotransducers at a load voltage of 15 V_{pp} produced a consistent separation distance, close to the theoretical separation distance of the acoustic pressure nodes (0.35 mm, black dashed line). Data was collected from three separate image for each group, with all datasets shown.

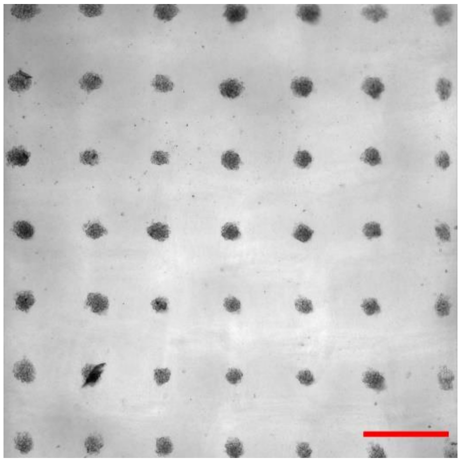

Supplementary Figure 10. **Low magnification image of a patterned hydrogel. Representative bright field image of myoblasts acoustically patterned in a 5% (w/v) PEG norbornene hydrogel. The hydrogel was crosslinked after 5 min of patterning, swollen overnight in PBS and then imaged. Scale bar = 500 µm.**

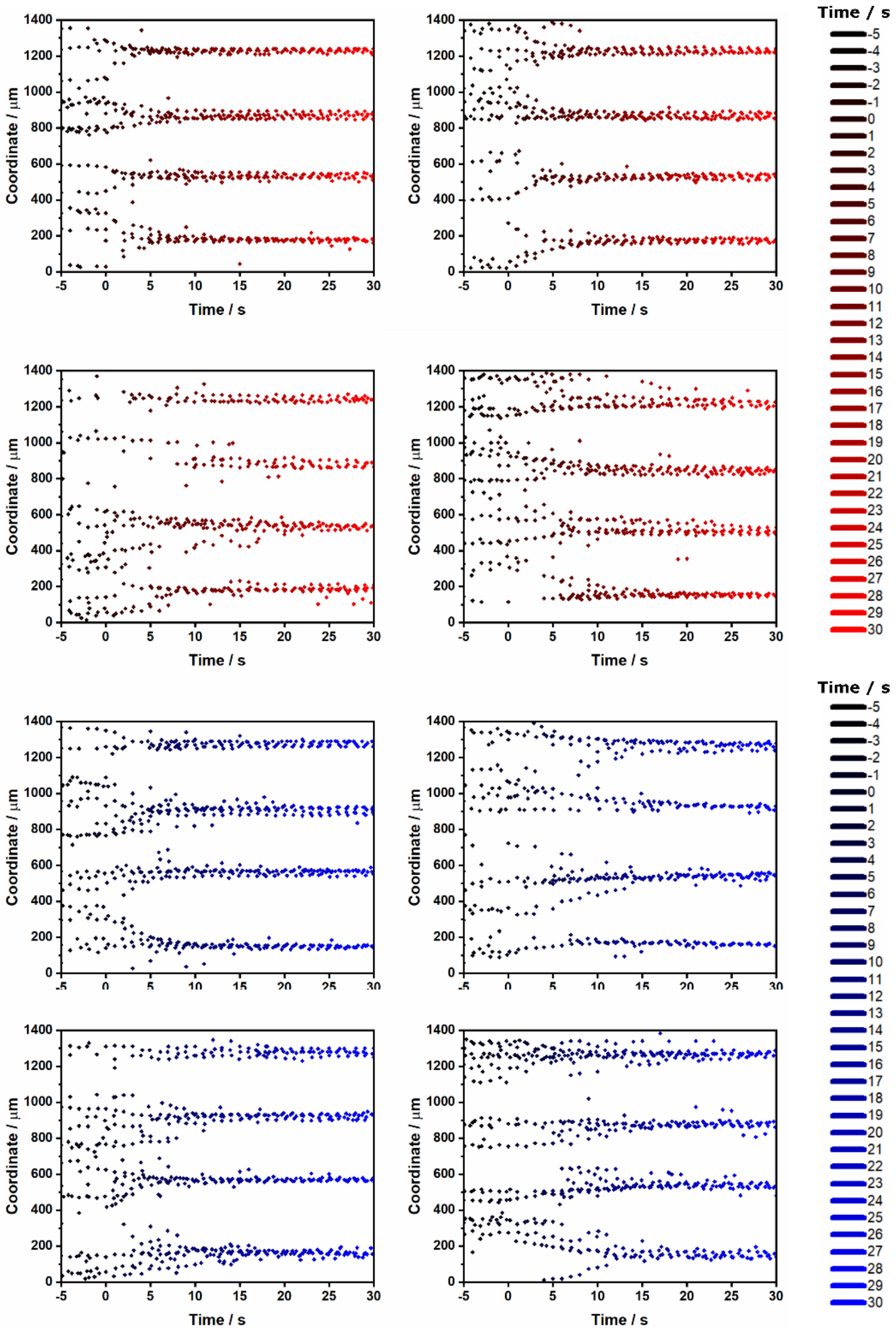

Supplementary Figure 11. Analysis of cluster barycentre coordinates during acoustic cell patterning. The cluster barycentre x coordinates (red) and y coordinates (blue) were plotted as a function of time. Data was collected from four separate videos of patterning, with all datasets shown.

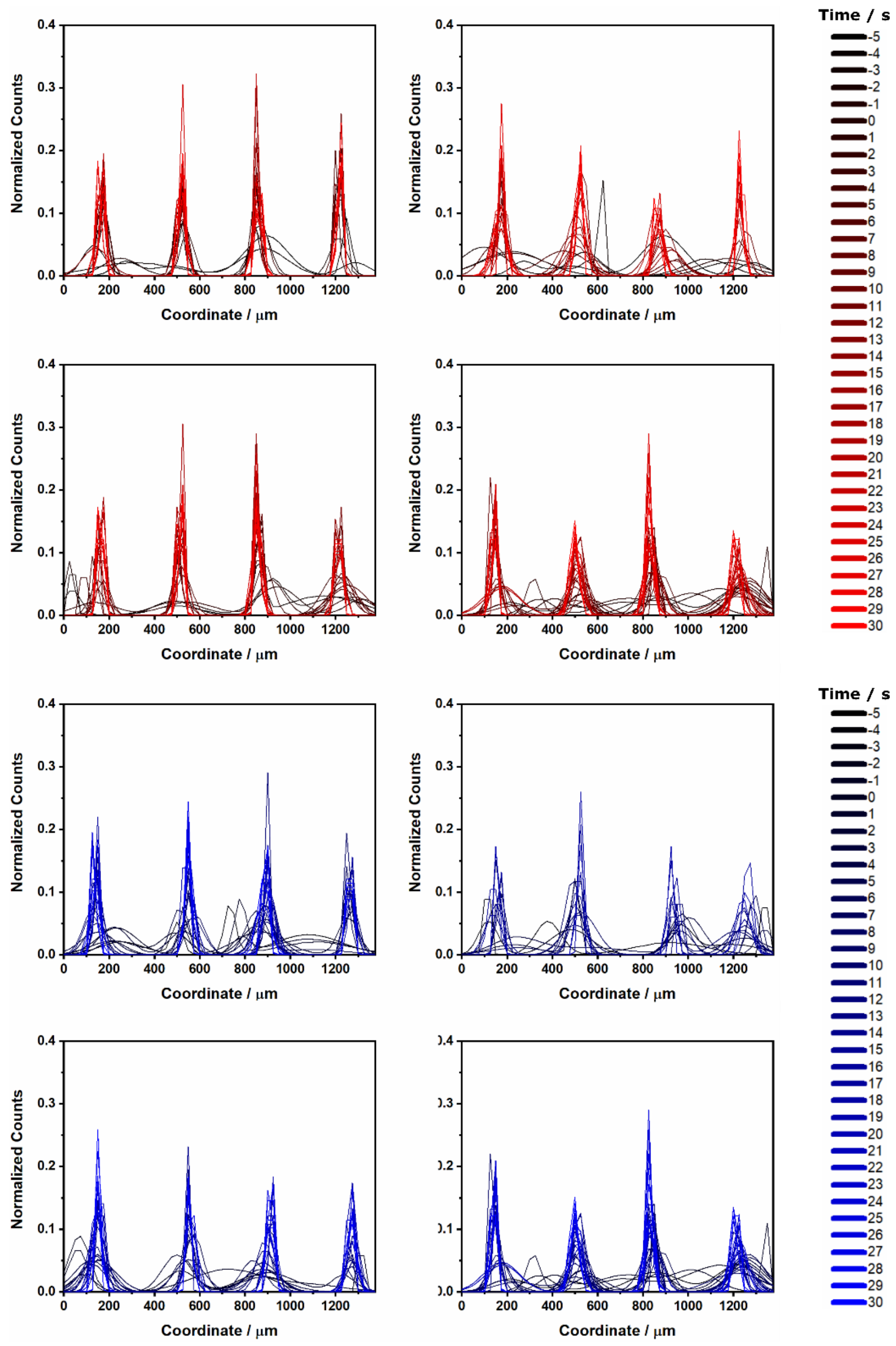

Supplementary Figure 12. Fitting of cluster barycentre distribution during acoustic cell patterning. Tetramodal Gaussian distribution mixture analysis curves based on the Expectation Maximization algorithm of the cluster barycentre histograms at different time points during patterning (see Supplementary Figure 8). This showed a transition over time from a reasonably uniform distribution to a defined periodic profile, for both the x coordinate (red) and y coordinate (blue) fits. Data was collected from four separate videos of patterning and temporally binned at 1 s intervals, with all datasets shown.

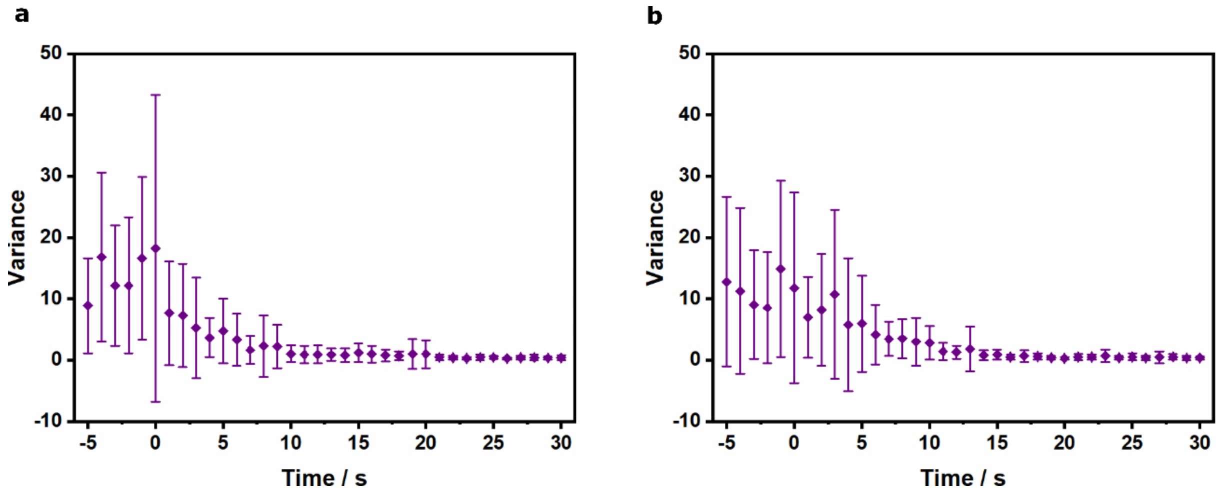

Supplementary Figure 13. Variance of cluster barycentre distribution fits during acoustic cell patterning. Histogram plots of the cluster barycentre coordinates were fitted to tetramodal distributions (see Supplementary Figure 9), with the variance of the Gaussian fits expressed as a function of patterning time. Initially, the random distribution of cluster barycentres in the unpatterned system produced broad curves with relatively high variance. After the field was turned on (from t = 0 s), the cell patterning produced periodic arrays with increasingly sharp curves and low variance. This trend was observed in fitted data from both (a) the x coordinates and (b) the y coordinates. Data plotted as mean ± standard deviation from tetramodal fits of four separate videos of patterning.

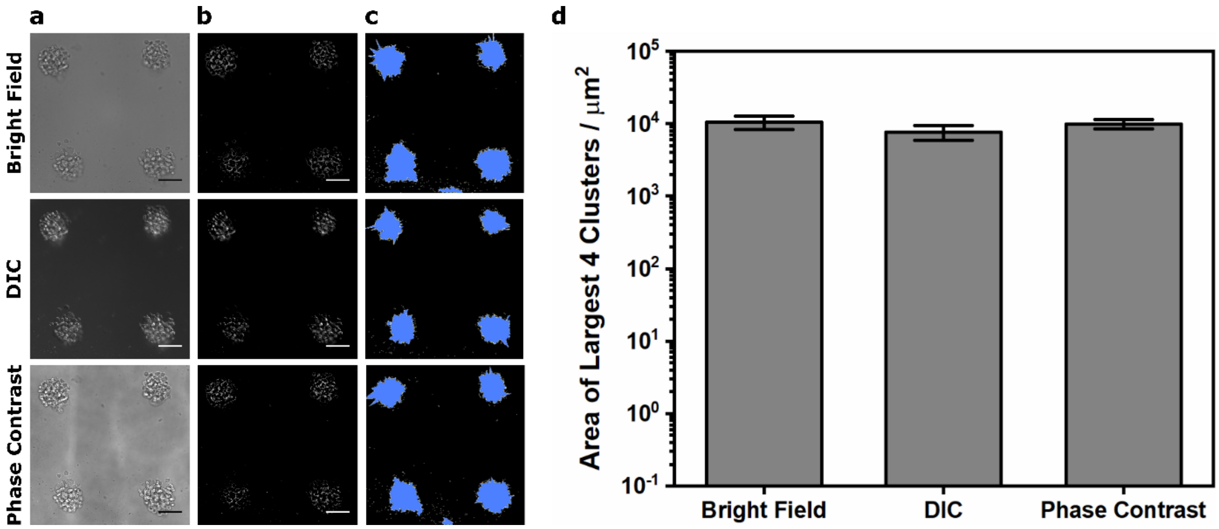

Supplementary Figure 14: Voronoï tessellation analysis of unlabelled cells. (a) Acoustically-patterned cells were imaged using bright field, differential interference contrast (DIC) and phase contrast microscopy. Scale bars = 100 µm. (b) The bright field and phase contrast images were inverted, and the minimum brightness level was raised for all three images to remove background noise. Scale bars = 100 µm. (c) Following Voronoï tessellation, clusters could be readily identified (blue). (d) The area of the largest four clusters were analyzed and shown to be similar for each imaging method. Data shown is the mean and standard deviation of the largest four clusters from a single image.
